# Pyrazolo[4,3-*e*]tetrazolo[1,5-*b*][1,2,4]triazine Sulfonamides as Novel Potential Anticancer Agents: Apoptosis, Oxidative Stress, and Cell Cycle Analysis

**DOI:** 10.3390/ijms24108504

**Published:** 2023-05-09

**Authors:** Karol Bukowski, Beata Marciniak, Mateusz Kciuk, Somdutt Mujwar, Mariusz Mojzych, Renata Kontek

**Affiliations:** 1 Department of Molecular Biotechnology and Genetics, University of Lodz, 90-237 Lodz, Poland; karol.bukowski@edu.uni.lodz.pl (K.B.); beata.marciniak@biol.uni.lodz.pl (B.M.); mateusz.kciuk@edu.uni.lodz.pl (M.K.); 2 Doctoral School of Exact and Natural Sciences, University of Lodz, 90-237 Lodz, Poland; 3 Chitkara College of Pharmacy, Chitkara University, Rajpura 140401, Punjab, India; somduttmujwar@gmail.com; 4 Department of Chemistry, Siedlce University of Natural Sciences and Humanities, 08-110 Siedlce, Poland; mariusz.mojzych@uph.edu.pl

**Keywords:** pyrazolo[4,3-*e*]tetrazolo[1,5-*b*][1,2,4]triazine, sulfonamides, MM compounds, anticancer agents, cancer cells, cell cycle, apoptosis, oxidative stress, ROS, CDKs

## Abstract

The current study continues the evaluation of the anticancer potential of three de novo synthesized pyrazolo[4,3-*e*]tetrazolo[1,5-*b*][1,2,4]triazine sulfonamides—**MM129**, **MM130**, and **MM131**—against human cancer cells of HeLa, HCT 116, PC-3, and BxPC-3 lines. The pro-apoptotic activity of the investigated sulfonamides was shown by observations of changes in the mitochondrial transmembrane potential of the tested cells, externalization of phosphatidylserine on the cellular membrane surface, and cell morphology in microscopic imaging. The computational studies have shown that **MM129** exhibited the lowest binding energy values when docked against CDK enzymes. In addition, the highest stability was shown for complexes formed between MM129 and CDK5/8 enzymes. All examined compounds induced cell cycle arrest in the G0/G1 phase in the BxPC-3 and PC-3 cells and simultaneously caused the accumulation of cells in the S phase in the HCT 116 cells. In addition, the increase in the subG1 fraction was observed in PC-3 and HeLa cells. The application of a fluorescent H_2_DCFDA probe revealed the high pro-oxidative properties of the tested triazine derivatives, especially **MM131**. In conclusion, the obtained results suggest that **MM129**, **MM130**, and **MM131** exhibited strong pro-apoptotic properties towards investigated cells, mainly against the HeLa and HCT 116 cell lines, and high pro-oxidative potential as well. Moreover, it is suggested that the anticancer activity of the tested compounds may be associated with their ability to inhibit CDK enzymes activities.

## 1. Introduction

Nowadays, cancer is among the leading causes of death worldwide. Therefore, searching for new effective strategies for cancer treatment is among the most challenging tasks in medicine [1,2,3,4]. The evaluation of the pro-apoptotic properties of novel potential antineoplastic agents, as well as their impact on cell cycle phase distribution, seems to be crucial in modern drug design. Apoptosis is a highly regulated process that plays a fundamental role in eliminating unwanted or unnecessary cells. Apoptosis dysregulation is connected with autoimmune disorders, neurological diseases, and many types of cancer. Because apoptosis evasion is a hallmark of all types of cancer, inducing apoptosis in cells to limit their uncontrolled proliferation appears to be a feasible and highly successful strategy to combat many types of cancer [5,6].

Apoptosis is linked to morphological changes in cells. The progressive process involves changes in the orientation of membrane phospholipids, which culminates in the externalization of phosphatidylserine (PS) onto the surface of cellular membranes [7,8,9]. Another characteristic feature of apoptotic cells is the increased permeability of the mitochondrial membrane and the simultaneous decrease in mitochondrial transmembrane potential (Δ*Ψm*). At the end of the apoptosis process, cell shrinkage and nuclear chromatin condensation and fragmentation are observed [10,11,12].

Reactive oxygen species (ROS) play an important role in the pathophysiology of cancer. The excessive intracellular levels of ROS stimulate the growth factor-dependent pathways, increase oncogene activity, and oxidize enzymes, inducing genetic instability [13,14]. Additionally, high levels of ROS can generate DNA, protein, and lipid damage. The induction of excessive ROS in cells is the primary mechanism of the cytotoxic activity of many commonly used anticancer agents, including bleomycin or doxorubicin [15]. Therefore, the pro-oxidative activity of various antineoplastic agents may affect their genotoxicity, enhance their pro-apoptotic properties, and indirectly modulate the cell cycle progression [16].

The role of cell cycle checkpoints is to protect the DNA from the accumulation and propagation of genetic errors during cell division. A checkpoint can pause the cell cycle or induce programmed cell death (PCD) when there is a threat to genetic integrity, such as irreversible DNA damage [17]. Those checkpoints are primarily regulated by cyclin-dependent kinase inhibitors (CKIs), cyclin-dependent kinases (CDKs), and cyclins [18]. In response to environmental stressors that prevent the cell from dividing or when the G0 phase lasts too long, the G1/S checkpoint causes cell cycle arrest, allowing the cell to repair damaged DNA. In contrast, the activation of the G2/M restriction point is regulated by the TP53 and occurs in response to DNA damage leading to the elimination of any damaged and inaccurately duplicated cells. The last restriction point is the spindle checkpoint that occurs in the M phase and delays cell division to guarantee precise chromosome segregation [19,20]. Therefore, targeting cell cycle checkpoints seems to be a promising strategy for cancer treatment [21].

CDKs are involved in the control of transcription, cell growth, proliferation, and in the regulation of the cell cycle. Furthermore, they play a crucial role in the pathogenesis of cancers [22,23]. CDK1 interacts with cyclin B1, allowing the progression of the cell cycle through the G2 phase, while CDK2 is responsible for G1/S and S/G2 transition [23,24,25,26,27,28]. Moreover, CDK2 is involved in controlling proliferation, cell differentiation, adaptive immune response, and apoptosis. It also, together with CDK3, mediates either senescence or quiescence [22,29,30,31,32]. Overexpression of CDK1 and cyclin B, as well as CDK2 binding partner cyclins E and A, is frequently observed in many types of cancer, including lung, breast, prostate, and colorectal carcinomas [33,34,35,36,37,38,39,40]. The inhibition of the cyclin D-CDK4/6 dimer leads to cell cycle arrest in the G1 phase [23,41]. Interestingly, colorectal cancer pathogenesis is often associated with the amplification of genes encoding CDK4/6, CDK5, CDK8, or CDK9 or their increased activity [42,43,44,45,46,47,48,49,50]. CDK7 participates in the DNA repair process, allows S phase entry by phosphorylation of CDK2/cyclin E complex, and helps to activate CDK1/cyclin B complex, allowing cell cycle progression from the G2 to M phase [51]. Numerous studies have revealed that CDK8 is involved in the regulation of transcription and frequently participates in the development of cancer. Furthermore, it regulates the cancer cell stress response to chemotherapy and radiotherapy and promotes tumor cell invasion, metastasis, and drug resistance [52,53,54,55,56]. Additionally, CDK8 plays an essential role during TP53-dependent p21 transcriptional activation, required for cell cycle arrest as the response to DNA damage [57,58]. Silencing CDK genes in various types of cancer downregulates their proliferation and leads to cell death. Therefore, CDKs are an attractive target for the relatively new class of antineoplastic agents called CDK inhibitors [22,23].

**Figure 1:**
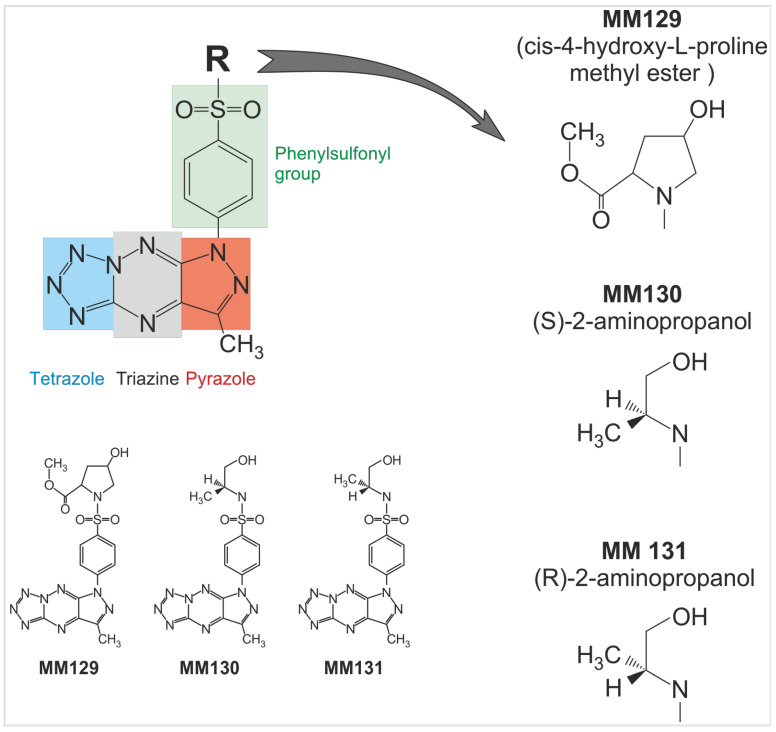
Chemical structure of the three investigated sulfonamides—**MM129**, **MM130**, and **MM131**.

Currently, several novel pyrazolo[4,3-*e*]tetrazolo[4,5-*b*][1,2,4]triazine derivatives are at different stages of the in vitro and in vivo investigations. In this paper, we present the anticancer properties of **MM129**, **MM130**, and **MM131** belonging to this group of structurally related compounds (Figure 1). Recent studies have shown that **MM129** may inhibit CDK2, AKT serine/threonine-protein kinase, PD-1/PD-L1, Bruton’s tyrosine kinase (BTK), phosphoinositide-3-kinase (PI3K) and mammalian target of rapamycin (mTOR) [59,60]. Furthermore, some studies suggest that the **MM129** may be a potentially safe and well-tolerated chemotherapeutic agent in colon cancer treatment [61]. Interestingly, **MM129** is characterized by a similar chemical structure compared to seliciclib (roscovitine). This antineoplastic agent is the first selective oral CDK inhibitor that entered clinical trials [62]. 

Our previous paper described the synthesis of **MM129**, **MM130**, and **MM131** compounds. Furthermore, the high cyto- and genotoxic properties of the investigated triazine derivatives against the HeLa, HCT 116, PC-3, and BxPC-3 cancer cell lines have been demonstrated. Importantly, the investigated compounds were noticeably less cytotoxic toward normal cells—human foreskin fibroblast (Hs27) and human peripheral blood mononuclear cells (PBMCs). The IC_50_ values obtained in the MTT assay were shown in the Supplementary Material (Table S1) [63]. Therefore, we decided to investigate the pro-oxidative and pro-apoptotic properties of three novel derivatives of pyrazolo-triazine sulfonamides **MM129**, **MM130**, **MM131** against four adherent human cancer cell lines—HeLa, HCT 116, PC-3, and BxPC-3. The assessment of the effect of these compounds on the cell cycle phase distribution was also evaluated. Moreover, we conducted computational studies (molecular docking and molecular dynamics techniques) to confirm our results from the cell cycle analysis with the potential ability of examined sulfonamides to inhibit CDKs. 

## 2. Results

### 2.1. Flow Cytometry Assessment of Annexin V Binding

**Figure 2:**
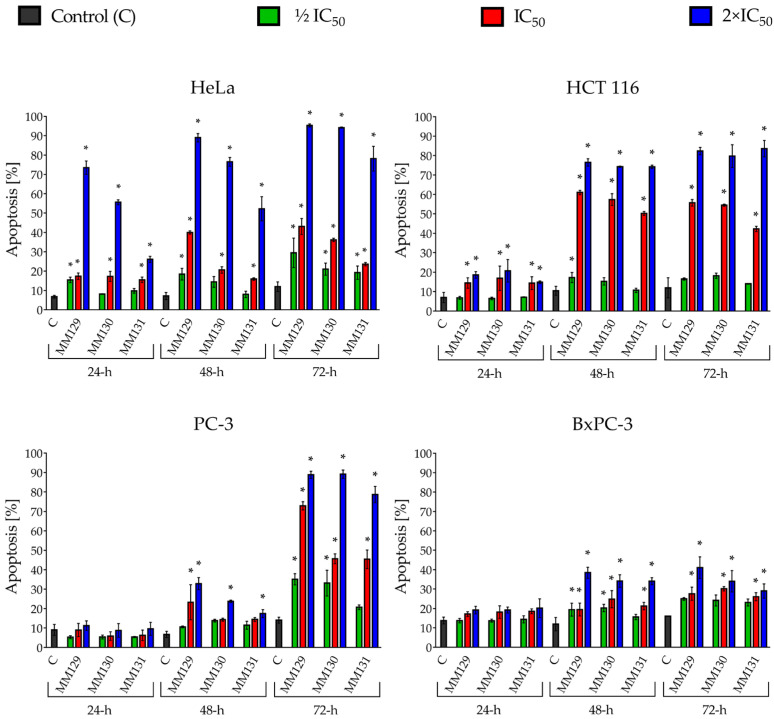
Percentage of apoptotic cells in the examined human cancer cell lines (Hela, HCT 116, PC-3, and BxPC-3) following 24 h, 48 h, and 72 h treatment with **MM129**, **MM130**, and **MM131** used at ½ IC_50_, IC_50_, and 2×IC_50_ and determined using FITC Annexin V Apoptosis Detection Kit I. Data are shown as the mean ± SD. * *p* < 0.05 indicates a statistically significant difference compared to the negative control. *N* = 1 × 10^4^.

Figure 2 shows the apoptosis induction in cancer cells following their 24 h, 48 h, and 72 h incubation with **MM129**, **MM130**, and **MM131** compounds. Studied pyrazole-triazine derivatives induced time and concentration-dependent increase in the fraction of apoptotic cells. Among the examined cell lines, Hela cells showed the highest sensitivity for the pro-apoptotic activity of MM compounds, even after the 24 h incubation, where the apoptosis induction for the IC_50_ and 2×IC_50_ compounds was significantly higher compared to the untreated cells (6.73% for the control group vs. 15.5–73.47% for experimental samples). Moreover, an increase in the percentage of apoptotic HCT 116 cells after their 24 h exposure to sulfonamides at the IC_50_ and 2×IC_50_ was noticed (6.92% for the untreated samples vs. 14.33–20.67% for experimental groups). 

All triazine derivatives investigated at 2×IC_50_ induced apoptosis of cancer cells after the 48 h incubation (HeLa = 52.23–88.97%; HCT 116 = 74.23–76.47%; BxPC-3 = 34.05–38.4%; PC-3 = 17.43–32.83%). The most profound proapoptotic effect was noticed after the 72 h treatment of cells with sulfonamides. MM compounds at the IC_50_ and 2×IC_50_ induced significant pro-apoptotic activity in all tested cancer cell types (23.6–95.3% of apoptotic cells following treatment). 

Among the investigated compounds, **MM129** exhibited the highest pro-apoptotic activity. **MM129** at ½ IC_50_ was the only one that induced apoptosis in HeLa cells following the 24 h of incubation. In addition, it was also the most effective toward PC-3 cells, where significant changes in the apoptotic cell fraction after the 48 h of exposure were observed even at the IC_50_. 

The representative flow cytometry dot-plots for all cancer cell lines were demonstrated in Supplementary Materials (Figures S1–S12). The fraction of necrotic cells in all experimental series has not exceeded 15%, even after 72 h exposure of cells to the tested compounds in their highest concentrations (2×IC_50_).

### 2.2. Dual Acridine Orange/Ethidium Bromide (AO/EB) Fluorescent Staining

**Figure 3:**
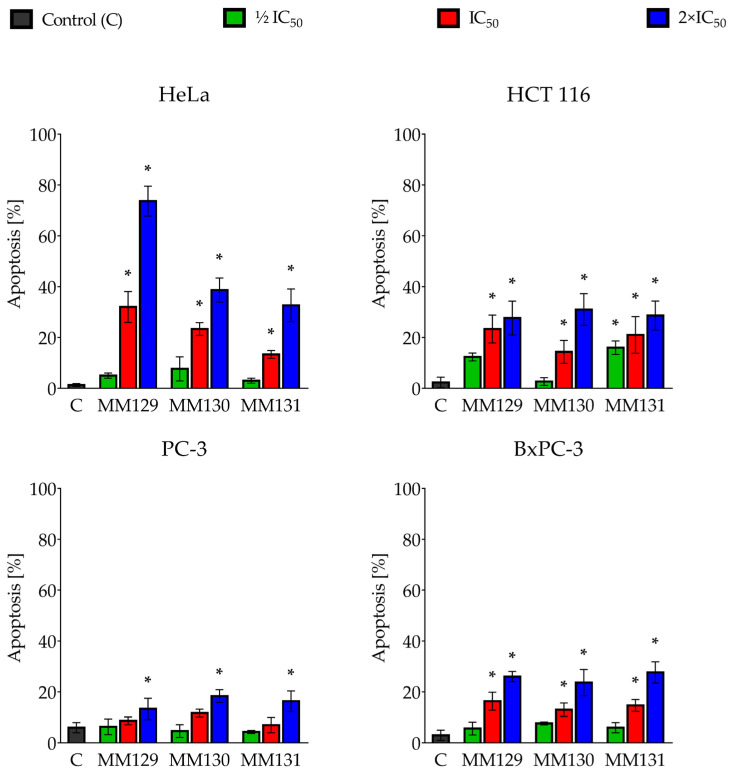
Apoptosis induction in human cancer lines (Hela, HCT 116, PC-3, and BxPC-3) treated for 48 h with **MM129**, **MM130**, and **MM131** at ½ IC_50_, IC_50,_ and 2×IC_50_ (obtained in the MTT assay). The results were based on AO/EB double staining, which allowed the distinguishing of viable, apoptotic, and necrotic cells. Data are shown as the mean percentage of apoptotic cells ± SD values. Significant differences (*p* < 0.05) compared to the negative control (*). *N* = 200.

Figure 3 represents apoptosis induction in cancer cells (HeLa, HCT 116, PC-3, and BxPC-3 cell lines) following their 48 h of exposure to **MM** compounds estimated by AO/EB double staining. The obtained results showed that the percentage of apoptotic cells increased in a concentration-dependent manner in all examined cancer cell types. Significant differences (*p* < 0.05) compared with vehicle control were observed for all examined compounds at 2×IC_50_ in all cancer cells. Moreover, all the investigated triazine derivatives induced apoptosis at the IC_50_ in HeLa, HCT 116, and BxPC-3 cells. **MM129** at 2×IC_50_ presented the strongest pro-apoptotic effect compared to other MM compounds in the HeLa cell line (73.7 ± 5.86% for **MM129** vs. 38.67 ± 4.73% and 32.67 ± 6.43% for **MM130** and **MM131**, respectively).

**Figure 4:**
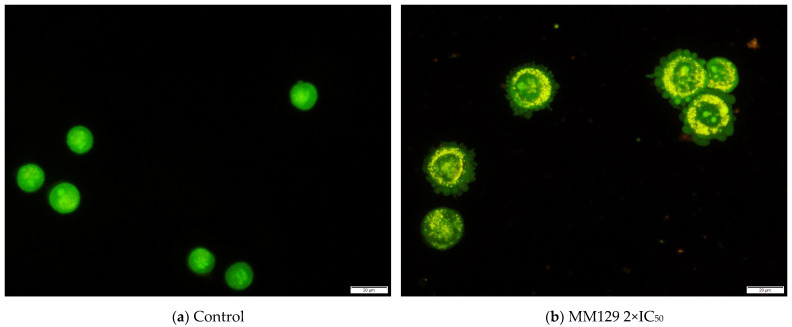
Representative images of HeLa cells obtained after AO/EB double staining following their 48 h incubation with **MM129** at 2×IC_50_. (**a**) Negative control group (control cells)—rounded cells with a uniformly distributed green-fluorescent nucleus; (**b**) experimental group (apoptotic cells)—yellow-orange fluorescence and characteristic cells’ membrane blebbing (apoptotic bodies formation). The scale bar is 20 μm. Magnification 40×.

The results obtained in OA/EB staining confirmed the presence of morphological changes characteristic of apoptotic cells, such as shrinking and condensation of chromatin after exposure to the tested sulfonamides. Figure 4 shows the exemplary images from this experiment. 

### 2.3. Mitochondrial Membrane Potential (ΔΨm)

**Figure 5:**
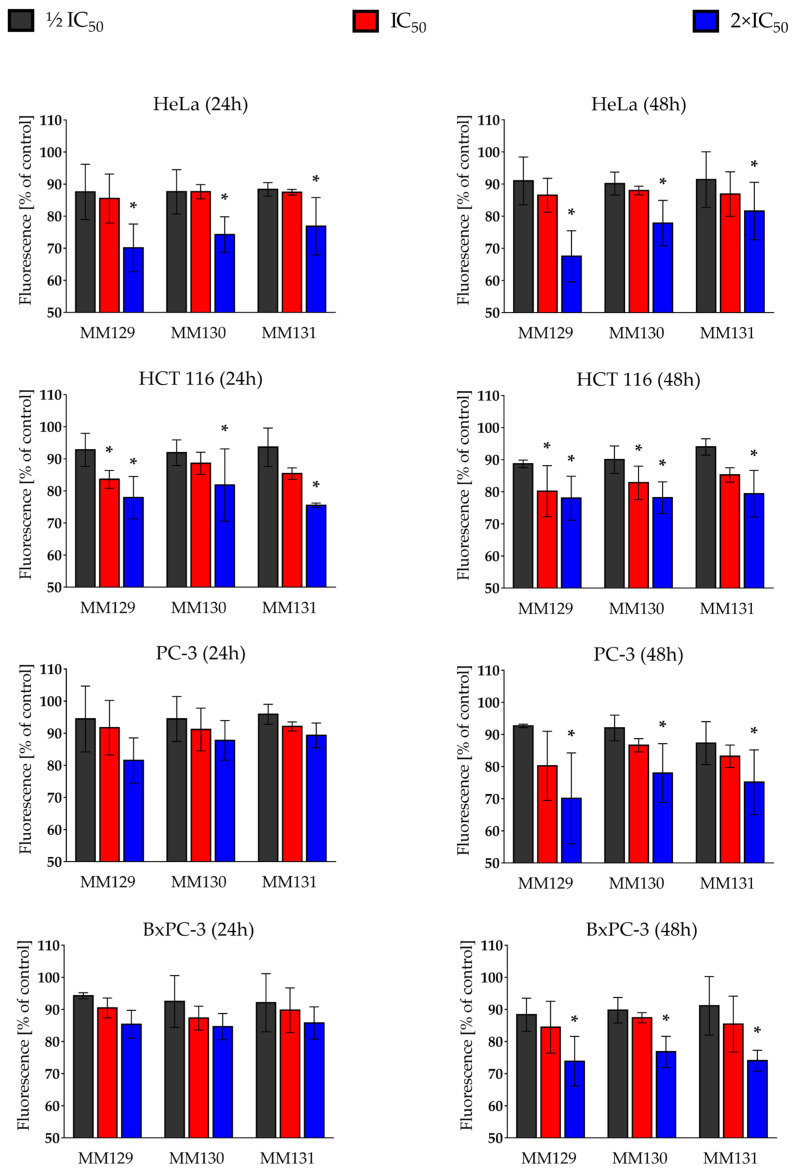
Changes in mitochondrial membrane potential (Δ*Ψm*) in human cancer lines (Hela, HCT 116, PC-3, and BxPC-3) after 24 h and 48 h exposure to **MM129**, **MM130**, and **MM131** at ½ IC_50_, IC_50_, and 2×IC_50_ (obtained in the MTT assay) using MitoTracker Red CMXRos. Data are presented as the mean ± SD relative to the control group normalized to 100%. * *p* < 0.05 indicates a statistically significant difference compared to the negative control.

Figure 5 presents changes in the Δ*Ψm* induced by **MM129**, **MM130**, and **MM131** following 24 h and 48 h incubation time. Exposure to the investigated sulfonamides resulted in concentration-dependent depolarization of the inner mitochondrial membrane in cells from all tested cancer lines. 

After 48 h of exposure to the studied sulfonamides at their highest applied concentration (2×IC_50_), significant alterations in Δ*Ψm* were observed in all tested types of cancer cells. Following the 24 h of incubation time, a significant disturbance of Δ*Ψm* was noticed only in Hela and HCT 116 cells. The most profound decrease in the Δ*Ψm* was observed in the HeLa cell line after 48 h exposure to **MM129** (67.53 ± 7.94% of control value). 

### 2.4. Determination of Intracellular ROS Level Using H_2_DCFDA

**Figure 6:**
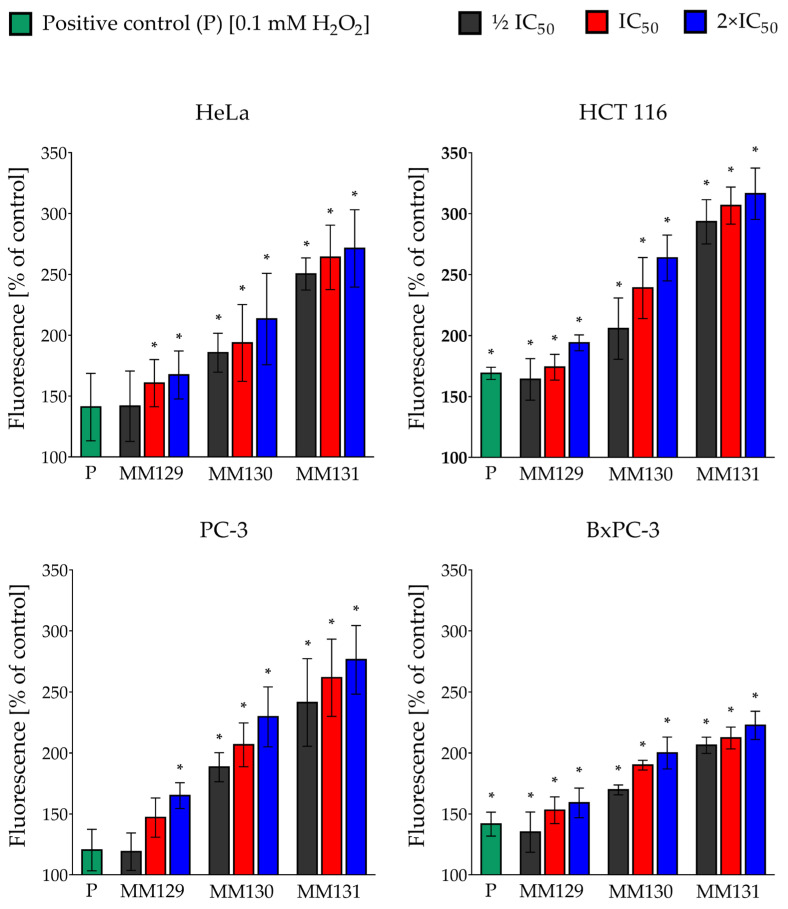
Changes in intracellular ROS level in the human cancer cell lines (HeLa, HCT 116, PC-3, and BxPC-3) following their 2 h treatment with **MM129**, **MM130**, and **MM131** at ½ IC_50_, IC_50_, and 2×IC_50_ (values obtained in the MTT assay). ROS level is expressed as the mean percentage of fluorescence intensity of H_2_DCFDA relative to control ± SD. Significant differences (*p* < 0.05) compared with negative control (*).

Figure 6 presents the changes in ROS level observed after 2 h exposure of the tested cells to MM compounds. Examined sulfonamides induced concentration-dependent increase in ROS level in all tested cancer cell types. All studied compounds caused significant changes in HCT 116 and BxPC-3 cancer cells when compared to the negative control, even at the lowest concentrations applied. The same results were obtained for HeLa and PC-3 cells except for **MM129**, which significantly increased intracellular ROS level only in its higher concentrations (IC_50_ and 2×IC_50_ for the Hela cell line and 2×IC_50_ for the PC-3 cell line). The administration of **MM131** resulted in the most significant elevation of reactive oxygen species (ROS) levels across all the tested cancer cell lines, with an observed increase ranging from 206.3% to 316.3% compared to control conditions.

### 2.5. Cell Cycle Analysis

**Figure 7:**
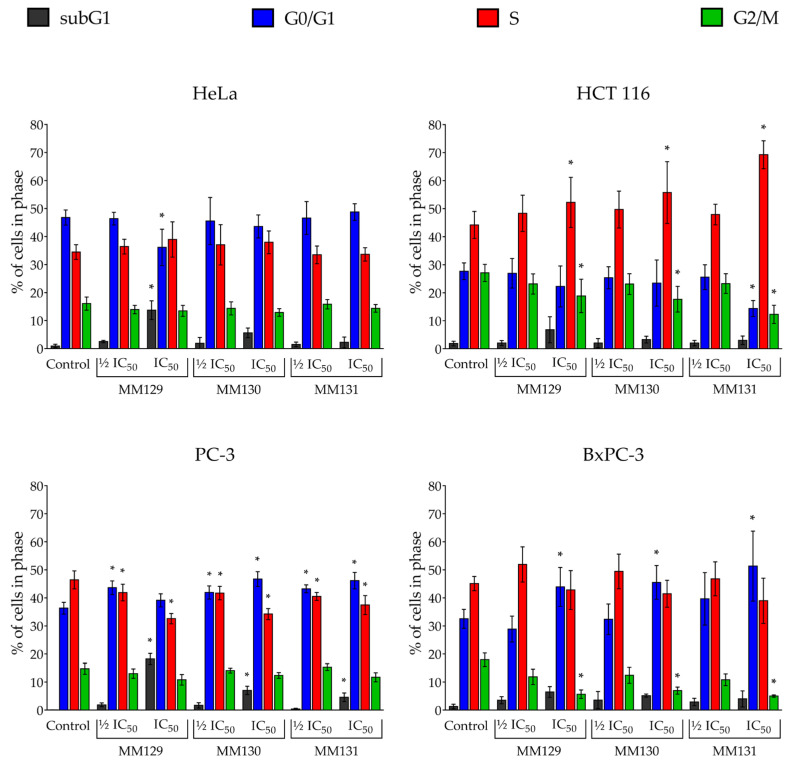
Cell cycle phase distribution of HeLa, HCT 116, PC-3, and BxPC-3 cancer cells after 24 h incubation with **MM129**, **MM130,** and **MM131** at ½ IC_50_ and IC_50_ are shown as the mean percentage of cells of a particular cell cycle phase ± SD. Significant differences (*p* < 0.05) compared with control (*). *N* = 1 × 10^4^.

Figure 7 demonstrates the cell cycle phase distribution of tested cancer cells after 24 h exposure to MM compounds. Of the sulfonamides tested, only **MM129** at the IC_50_ concentration significantly affected cell cycle progression in HeLa cells. The incubation of the tested cells with **MM129** caused an increase in the percentage (%) of cells in the subG1 phase, and simultaneous decrease in the number of cells (%) in the G0/G1 phase. In the case of HCT 116 cells, an accumulation of cells in the S phase and a reduction in the proportion of cells in the G2/M phase after treatment with triazine derivatives at the IC_50_ was observed. The cell cycle distribution of PC-3 cells showed that all tested MM compounds increased the number of cells (%) in the G0/G1 and subG1 phases and caused a decrease in the % of cells in the S phase. In contrast, an increase in the number of cells in the G0/G1 phase and a reduction in cells in the G2/M phase was observed following the exposure of BxPC-3 cells to examined sulfonamides at the IC_50_. The representative flow cytometry histograms for the tested cell lines were demonstrated in Supplementary Materials (Figure S13).

### 2.6. Molecular Docking

**MM** compounds/CDK complexes were analyzed according to their binding energy (kcal/mol) obtained during the molecular docking procedure as presented in Table 1.

**MM129** exhibited the lowest binding energy values when docked against CDK enzymes. Therefore, it showed the best binding potential among the MM compounds (except for the docking of **MM131** with CDK6). **MM129** showed more profound binding potential compared with reference ligands of CDK enzymes (except for CDK9). The highest binding affinities were observed for CDK4 and CKD7 (binding energy of −9.48 and −9.84, respectively). However, we have decided to assess the molecular stability of **MM129** with all the investigated CDK enzymes.

### 2.7. Molecular Dynamics Simulations

Sufficient stability of the **MM129**/CKDs complexes was observed during molecular dynamics simulation. The highest stability based on the observed parameters such as root mean square deviation (RMSD), root mean square fluctuation (RMSF), protein–ligand contacts, and conserved secondary structure were shown for complexes formed between **MM129** and CDK5/8 enzymes. Thus, it has been supposed that **MM129** executes the anticancer effect via synergistic targeting of both CDK5 and CDK8 enzymes.

The target enzyme CDK5 has 276 residues distributed in a macromolecular chain consisting of 2225 heavy atoms out of a total of 4505 atoms. The macromolecular target has 35% of secondary structures in the form of 27% of alpha helices and 8% of beta-strands, which were found to be conserved during the simulation process. **MM129** possesses 32 heavy atoms out of a total of 51 atoms. Dynamic simulation of the macromolecular complex of **MM129** against the CDK5 target has clearly shown that the RMSD for the fluctuation of the protein backbone was in-between 1.2–2.0 Å, which is well within the acceptable range. Similarly, the ligand **MM129** showed some initial adjustment up to 5 ns within the active site to achieve the stable conformation followed by its stabilized vibrations within the range of 3.6–4.8 Å. Afterward, the complexed ligand attained the most stable conformation and remained stable throughout the simulation process. The RMSF of the macromolecular backbone was found to be well within the range of 0.4–2.0 Å throughout the simulation process. Macromolecular residues such as Ile10, Tyr15, Gly16, Val18, Cys83, Asp86, Lys128, Gln130, Asn131, Leu133, and Asn144 of CDK5 were found to interact with the ligand **MM129** throughout the simulation process.

**Figure 8:**
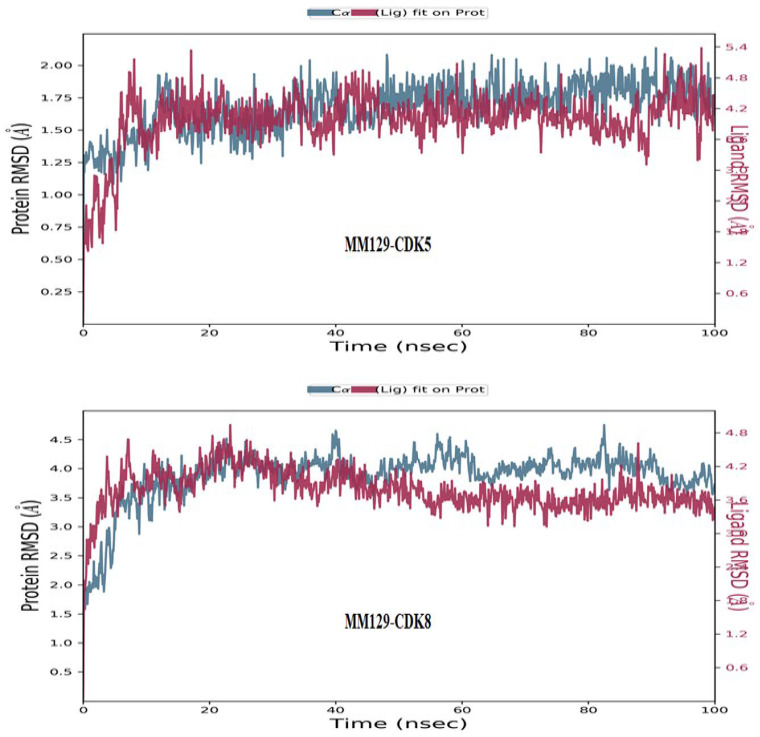
RMSD of the Cα backbone of human CDK5-**MM129** and CDK8-**MM129** receptor–ligand complex observed during the 100 ns of MD simulation.

The target enzyme CDK8 has 346 residues distributed in a macromolecular chain consisting of 2855 heavy atoms out of a total of 5716 atoms. The macromolecular target has 35% of secondary structures in the form of 25% of alpha helices and 10% of beta-strands, which were found to be conserved during the simulation process. Dynamic simulation of the macromolecular complex of **MM129** against the CDK8 target clearly showed that the RMSD for the fluctuation of the protein backbone was in-between 2.5–4.0 Å, which is well within the acceptable range. Similarly, the ligand **MM129** showed stable conformation within the range of 3.0–4.2 Å and remained stable throughout the simulation process. The RMSF of the macromolecular backbone was found to be well within the range of 0.6–2.4 Å except for some terminal residues throughout the simulation process. Macromolecular residues, such as Val27, Arg29, Thr31, Lys52, Phe97, Asp98, Ala100, Asp103, Trp105, Lys153, Ala155, Leu158, and Asp173 of CDK8 were found to interact with the ligand **MM129** throughout the simulation process. The RMSD of the macromolecular complex of **MM129** against CDK5 and CDK8 observed during the 100 ns of the MD simulation is displayed in Figure 8.

## 3. Discussion

In the current study, we have demonstrated the pro-apoptotic and pro-oxidative activity of **MM129**, **MM130**, and **MM131** towards Hela, HCT 116, PC-3, and BxPC-3 cancer cells. Furthermore, our research included computational studies and analysis of cell cycle distribution in the tested cells after 24 h exposure to **MM129**, **MM130**, and **MM131**. 

The obtained results of the Annexin V binding assay showed the pro-apoptotic activity of examined sulfonamides towards the HeLa, HCT 116, PC-3, and BxPC-3 cancer cell lines (Figure 2). Only the HeLa and HCT 116 cell lines showed significant changes in the apoptosis prevalence following 24 h exposure to the investigated compounds. A high number of apoptotic cells was detected following the treatment of the HeLa cells with **MM129** and **MM130** (73.47 ± 3.48% and 55.63 ± 1.27% of apoptotic cells at 2×IC_50_, respectively. Following 48 h incubation, all MM compounds demonstrated an enhancement in apoptotic rates across all the cancer cell lines when administered at their highest concentration (2×IC_50_). However, the most noticeable effect was observed for the HCT 116 and HeLa cells. The highest apoptotic response to the investigated triazine derivatives was observed after 72 h incubation of the tested cancer cell lines. The high apoptotic properties of **MM129** and **MM131** towards other cancer cells (HT-29 and DLD-1) were confirmed by other researchers [60,64]. 

During microscopic analysis typical morphological features of apoptosis, including chromatin condensation, cell shrinkage, nuclear fragmentation, and membrane blebbing, were noticed following the treatment of cells with MM compounds (Figure 4). Furthermore, a quantitative analysis of the AO/EB double-stained cells confirmed the pro-apoptotic potential of examined sulfonamides observed in the Annexin V binding assay (Figure 3). A significant increase in the number of apoptotic cells in all the cancer cell lines was noticed after 48 h incubation with investigated compounds at their highest concentration. Similarly to the Annexin V assay, the results of AO/EB revealed that HeLa was the most sensitive cell line to the pro-apoptotic activity of the compounds. In addition, the results of the AO/EB dual staining confirmed the pro-apoptotic activity of **MM131** towards DLD-1 and HT-29 cancer cells [60]. Furthermore, a similar pattern was observed in the case of other triazine derivatives against several cancer cell lines. 

In the present work, all studied sulfonamides depleted Δ*Ψm* in all the evaluated cell lines following 48 h of treatment. Additionally, a decrease in Δ*Ψm* was observed in the HeLa and HCT 116 cells after 24 h exposure to MM compounds in their highest concentration (Figure 5). The results of Hermanowicz et al. [64] also showed the ability of **MM129** to decrease the Δ*Ψm* in the DLD-1 and HT-29 cells. Moreover, the literature data have shown a similar activity of other MM compounds towards the different cancer cell lines [65,66]. According to the study of Ly et al. [67], loss of Δ*Ψm* may be an early event of the apoptotic-signaling pathway. However, Δ*Ψm* reduction may also be a consequence of apoptosis when it is triggered by the opening of the mitochondrial permeability transition pore (PTP) [67]. As mentioned above, the studies have indicated that the reduction of Δ*Ψm* is the effect of the opening of PTP. In contrast, other research works have suggested the opposite process—the loss of Δ*Ψm* is responsible for the opening of PTP [68]. Undoubtedly, loss of the Δ*Ψm* and opening of the PTP are associated with the release of pro-apoptotic proteins, such as cytochrome c, apoptosis-inducing factor (AIF), and procaspase-9. As a consequence, this leads to a circle of self-perpetuating apoptotic changes [67,69,70]. 

Moreover, the assessment of the pro-oxidative properties of examined compounds was conducted. All the investigated sulfonamides induced a concentration-dependent increase in ROS formation in all investigated cancer cell types following 2 h exposure (Figure 6). ROS accumulation may cause DNA strand break formation, mitochondrial DNA lesions, and degradation of mitochondrial DNA [16]. The high pro-oxidative activity of tested triazine derivatives is consistent with the data obtained from our previous studies, where examined compounds exhibited high genotoxicity against the same cancer cell lines. Similarly to the genotoxicity studies, **MM131** showed the most potent pro-oxidative potential among the tested compounds [63]. Furthermore, research has shown that the accumulation of ROS-related damages in lipids, proteins, and DNA may be responsible for the suppression of tumor cell proliferation and growth via cell cycle arrest and apoptosis. [71,72]. Literature data have suggested that raised cytosolic calcium ion levels and increased formation of ROS cause a decrease in electrolyte transport across the mitochondrial membrane, leading to the opening of PTP and collapse of Δ*Ψm* [68,73,74,75]. As a consequence, various pro-apoptotic molecules are released, activating the mitochondrial apoptosis pathway [15,73,76,77,78,79,80,81,82,83,84,85,86]. In addition, ROS can also enhance the death receptor-mediated apoptosis pathway by downregulation the FLICE-inhibitory protein (c-FLIP) half-life [87]. Moreover, ROS may bind to death receptors containing the death domain (DD), and thus initiate the extrinsic pathway of apoptosis through interaction with other adaptor proteins with DDs [88,89]. In addition, the activation of apoptosis by ROS is the result of mitochondrial damage and activation of poly(ADP-ribose) polymerase (PARP) and apoptosis signal-regulating kinase 1 (ASK1) [90]. Therefore, we conclude that the ROS formation observed in the tested cancer cells after the incubation with examined compounds may be the cause for the further loss of Δ*Ψm*. 

The cell cycle distribution of PC-3 cells showed a significant increase in the proportion of the cells in the G0/G1 phase and a reduction in the number of cells (%) in the S phase after the incubation with MM compounds. Similar results were obtained by Hermanowicz et al. [59] who treated the HT-29 and DLD-1 cells with **MM129**. A different response was observed in the BxPC-3 cell line. The incubation with triazine derivatives at the IC_50_ concentration caused an accumulation of cells in the G0/G1 phase and a decrease in the proportion of the cells in the G2/M phase. A similar reduction in the number [%] of cells in the G2/M phase was observed following treatment of HCT 116 cells with the investigated sulfonamides at the IC_50_. However, after incubation with the investigated compounds, HCT 116 cells showed an opposing trend in the distribution of cells in the S phase compared to PC-3 cells. All the tested compounds in at the IC_50_ increased the percentage of the cells in the S phase. Gornowicz et al. [60] observed a similar activity of MM131 in other colorectal cancer cell lines (HT-29 and DLD-1 cells), where this compound also increased the proportion of the cells in the S phase. The highest pro-apoptotic activity measured as a proportion of the cells in the subG1 phase in HeLa and PC-3 cells was noticed for **MM129** compound (Figure 7). Interestingly, the varied activity of the same antineoplastic drug against a diversity of cancer cell lines is consistent with earlier research findings. As an example, 5-fluorouracil (5-FU) increased the accumulation of cells in the G0/G1 phase in gastric cancer cells AGS and some colorectal cancer (CRC) cell lines, such as HT-29 and DLD-1 while also arresting oral cancer cells HSC-4 and colon cancer cells SW620 in the S phase [60,91,92,93]. Our previous research has shown that all examined triazine derivatives are highly genotoxic toward the tested cell lines [63]. Interestingly, the most noticeable increase in the number of cells in the S phase in HCT 116 cells was observed in the case of **MM131**. This is consistent with our previous results, where **MM131** induced the highest levels of DNA damage among the tested sulfonamides [63]. In turn, the elongated G0/G1 phase also provides the cell more time to repair DNA damage, preventing the it from entering the S phase. The genotoxic study of the investigated triazine derivatives has presented their high genotoxic activity not only against HCT 116 but also towards PC-3 and BxPC-3 cells [63]. Thus, the cell cycle arrest in the G0/G1 phase in PC-3 and BxPC-3 cells after the incubation with MM compounds may also be the effect of the accumulation of DNA damage. 

Finally, our results of molecular docking have shown the high potential of **MM129**, as well as **MM130**, and **MM131** to bind with CDK enzymes. **MM129** exhibited the lowest binding energy values among examined sulfonamides, especially for CDK4 and CDK7 (Table 1). The results of molecular dynamics simulations confirmed sufficient stability of the **MM129**/CKDs complexes. The highest stability was observed for the associations of **MM129** with CDK5 and CDK8 enzymes. In the study of Hermanowicz et al. [59], an upregulation of TP53 and simultaneous downregulation of CDK2 was observed after the exposure of the HT-29 and DLD-1 cell lines to **MM129**. The cell cycle arrest in the G0/G1 phase was noticed in both CRC lines after the incubation with **MM129**. Thus, the change that was seen in those cells was most likely induced by an increase in TP53 expression in conjunction with a downregulation of CDK2 activity [59]. Therefore, we conclude that the interaction of investigated compounds with CDKs may be among the potential causes of observed disturbances in the cell cycle distribution in the tested cells.

## 4. Materials and Methods

### 4.1. Cell Culture and Chemicals

All the examined adherent human cancer cell lines, including HeLa (cervical cancer), HCT 116 (colorectal carcinoma), PC-3 (prostate cancer), and BxPC-3 (pancreatic adenocarcinoma), were purchased from American Type Culture Collection (ATCC^®^, Rockville, MD, USA). HCT 116 and BxPC-3 cells were cultivated in RPMI 1640 medium, while IMDM and DMEM-F12 media were used for the cultivation of the HeLa and PC-3 cell lines, respectively. Media and Trypsin/EDTA used for cell culture were supplied by Biowest (CytoGen, Zgierz, Poland). All media were supplemented with 1% (*v*/*v*) penicillin-streptomycin solution and 10% (*v*/*v*) fetal bovine serum (FBS) (Merck/Sigma Aldrich Chemical Co, Burlington, MA, USA). FITC Annexin V Apoptosis Detection Kit I was obtained from B.D. Biosciences (Franklin Lakes, NJ, USA). MitoTracker CMXRos was supplied by Invitrogen (Waltham, MA, USA). Buffered saline (PBS), ethanol, dimethyl sulfoxide (DMSO), acridine orange/ethidium bromide (AO/BE), 2′,7′-dichlorodihydrofluorescein diacetate (H_2_DCFDA), RNase A Solution, and propidium iodide (PI) were purchased from Merck/Sigma Aldrich Chemical Co (Burlington, MA, USA). 

Cancer cells were maintained in a humidified incubator at 37 °C in a 5% CO_2_ atmosphere and were systematically scanned for mycoplasma contamination. The proper number of cultivated cells was managed by their regular passaging at 90% confluence twice weekly using 0.025% trypsin/EDTA. Additionally, to maintain stable growth conditions for cell culture, the medium was replaced every 48 h. Each experiment was performed on cells from three independent passages and included a negative control (cells treated with DMSO at <0.5%). The concentrations for all experiments were based on the values obtained in the MTT assay after 72 h of cell incubation with examined compounds (½ IC_50_, IC_50_, and 2×IC_50_) [63].

### 4.2. Flow Cytometry Assessment of Annexin V Binding

Evaluation of the induction of apoptosis in the HeLa, HCT 116, PC-3, and BxPC-3 cell lines was performed using the FITC Annexin V Apoptosis Detection Kit I via flow cytometry analysis. The translocation of the PS on the outer side of the cell membrane was associated with PCD. Annexin V conjugated with fluorescein isothiocyanate (FITC) binds to PS, which allows the identification of apoptotic cells, while not membrane-permeable PI binds to the DNA of necrotic cells. Double staining identifies independently early and late apoptosis (cells stained only with FITC or with both dyes, respectively) [94].

Cancer cells were cultured for 24 h in controlled conditions (5% CO_2_; 37 °C) on 6-well plates, and then exposed to MM compounds in different concentrations (½ IC_50_, IC_50_, and 2×IC_50_) for 24 h, 48 h, or 72 h. After incubation, the cells were trypsinized and transferred to conical tubes, then washed twice with cold PBS. Following centrifuging (10 min, 4 °C, 1400 rpm), the cells were suspended in Annexin V-binding buffer and stained with PI and FITC-labelled Annexin V for 15 min at room temperature in the dark. Additional controls were used to set up compensation and quadrants were included (unstained cells and cells dyed only with FITC Annexin V or PI). Measurements were performed using a flow cytometer (LSR II, Becton Dickinson, San Jose, CA, USA) at the wavelength of 530 nm. The obtained data (10,000 events per sample) were analyzed in FlowJo 10.8.1 software.

### 4.3. Dual Acridine Orange/Ethidium Bromide (AO/EB) Fluorescent Staining

Acridine orange (AO) intercalates into the DNA of living cells after penetration of their cell membrane and accumulates in lysosomes, emitting green and red fluorescence, respectively. In contrast, ethidium bromide (EB) interacts only with the DNA and RNA of membrane-damaged cells, emitting red fluorescence. This method allows the observation of the development of apoptotic bodies and detects nuclear alterations, which are both associated with apoptosis. The morphological changes in chromatin condensation in the dyed nucleus, combined with different uptake of AO and EB, allows for distinguishing three categories of cells as follows: viable (green-stained nucleus uniformly distributed in the center of the cell), apoptotic (orange-stained cell nuclei with condensation and chromatin clumping; the presence of cell membrane blebbing indicating the apoptotic bodies formation) and necrotic (increased cell’s volume; uneven orange-red fluorescence without a sharp outline; cell near disintegration) [95,96]. 

The cells were seeded for 24 h in controlled conditions (5% CO_2_; 37 °C) on 12-well plates, and then were incubated with MM compounds in different concentrations (½ IC_50_, IC_50_, and 2×IC_50_) for 48 h. After being cultured, the cells were trypsinized, transferred to conical tubes, and then centrifuged (10 min, 4 °C, 1400 rpm). Afterward, the supernatant was removed, and the precipitate was diluted in 25 μL of PBS and transferred to the Eppendorf tube. The suspension was mixed with dual fluorescent staining solution (1 μL) containing 100 μg/mL AO and 100 μg/mL EB, transferred to glass slides, and then covered with a coverslip. The morphology of the cells was conducted, and 200 cells were counted within 10 min using a fluorescent microscope (Olympus BX60 F5, Olympus Optical Co., Ltd., Nagano, Japan) at 360 nm. 

### 4.4. Mitochondrial Membrane Potential (ΔΨm)

Disruption of mitochondrial membrane potential is among the earliest changes associated with the induction of apoptosis [10]. As a result of the increased permeability of the internal and external mitochondrial membrane, the mitochondrial proteins, such as apoptosis-inducing factor (AIF) and cytochrome c migrate into the cytosol leading to the activation of the intrinsic apoptosis pathway. MitoTracker CMXRos, which contains thiol-reactive chloromethyl moiety passively diffuses across the plasma membrane. Accumulation of fluorescence probe in active mitochondria allows the measurement of changes in the Δ*Ψm* of the tested cells [67,97]

Cancer cells were seeded for 24 h in controlled conditions (5% CO_2_; 37 °C) on 96-well plates and then were treated with investigated compounds in different concentrations (½ IC_50_, IC_50_, and 2×IC_50_) for 24 h or 48 h. Following incubation, the cells were washed with PBS and stained with the probe at the final concentration of 0.1 μM for 40 min at 37 °C in the dark. The transmembrane mitochondrial potential was measured using a microplate reader at the wavelength of 579/599 nm.

### 4.5. Determination of Intracellular ROS Level by H_2_DCFDA

ROS are associated with the regulation of apoptosis pathways mediated via endoplasmic reticulum (ER), death receptors, and mitochondria [69]. Accordingly to the protocol described previously by Ruiz-Leal and George [98], H_2_DCFDA was used to determine the level of intracellular ROS formation. H_2_DCFDA is a cell-permeable stain widely used for the quick and effective detection of total intracellular oxidants. The probe can diffuse across an intact cellular membrane and then is hydrolyzed to 2′,7′-dichlorodihydrofluorescein (H_2_DCF) by intracellular esterases active in living cells. Finally, ROS oxidate dichlorodihydrofluorescein to a highly fluorescent 2′,7′-dichlorofluorescein (DCF).

Cancer cells were cultured for 24 h in controlled conditions (5% CO_2_; 37 °C) on black 96-well plates. Afterward, the culture medium was replaced by H_2_DCFDA dissolved in PBS (final concentration was 20 mM), and the cells were incubated for 20 min in the dark. Following incubation, cell lines were exposed to examined sulfonamides in different concentrations (½ IC_50_, IC_50_, and 2×IC_50_). After 120 min of incubation, the fluorescence was measured using a microplate reader at the wavelength of 485/535 nm using the SpectraMax^®^ i3xMulti-Mode Detection Platform.

### 4.6. Cell Cycle

The effect of MM compounds on the cell cycle was determined by flow cytometry. The cell cycle distribution of a sample was estimated by staining the DNA with a fluorescent dye, such as PI, which binds to the DNA stoichiometrically. The analysis of the cellular DNA content frequency histograms reveals the differentiation of cells in the G0/G1, S, and G2/M phases. Furthermore, this approach allows the identification of aneuploid populations, as well as apoptotic cells with fractional DNA content (subG1) [99].

The cells were seeded for 24 h in controlled conditions (5% CO_2_; 37 °C) on 6-well plates, and then were incubated with MM compounds at ½ IC_50_ and IC_50_ for 24 h. After incubation, the cells were trypsinized and transferred to conical tubes, then washed with PBS. Following centrifuging (10 min, 4 °C, 1400 rpm), the cells were fixed with cold ethanol (70%), then washed with PBS. Afterward, the cells were treated with 10 ug/mL of RNase A solution and stained with 5 μg/mL of PI for 30 min at 37 °C in the dark. The measurements were performed using a flow cytometer (LSR II, Becton Dickinson) at the wavelength of 530 nm. The obtained data (10,000 events per sample) were analyzed in FlowJo 10.8.1 software.

### 4.7. Molecular Docking

In an attempt to explore the potential inhibitory effects of **MM129**, **MM130**, and **MM131** on CDK enzymes, we have performed molecular docking. MM compounds were sketched in ChemDraw 8.0 software and their three-dimensional structure was obtained following energy minimization using an MM2 force field. To get the ligands ready for molecular docking simulation, we used AutoDock Tools (The Scripps Research Institute, La Jolla, CA, USA) to find aromatic carbons and rotatable bonds, configure the automated torsion number, margin non-polar hydrogens, and add Gasteiger charges. 

Three-dimensional structures of CDK enzymes (CDK1 (PDB ID: 6gu6), CDK2 (PDB ID: 3bhu), CDK4 (PDB ID: 2w9z), CDK5 (PDB ID: 1unh), CDK6 (PDB ID: 6oqo), CDK7 (PDB ID: 1ua2), CDK8 (PDB ID: 6t41), and CDK9 (PDB ID: 3blq) were obtained from Protein Data Bank (PDB) https://www.rcsb.org/ (accessed on 1 January 2023). Using AutoDockTools, the macromolecules were assigned autodock atom type (AD4), and the Gasteiger charge was added and distributed along the macromolecule. The structures were saved in PDBQT file format. 

The grid box values were adjusted following the docking of reference ligands already complexed in the PDB structure after throughout observation of the drug’s conformational poses. The grid parameters for each CDK enzyme were saved in a grid parameter file (GPF). The Autogrid utility from the Autodock suite was used to create the additional map files required for running the molecular docking simulations (Table 2).

### 4.8. Molecular Dynamics Simulations

To validate the obtained docking outcome for the thermodynamic stability of the macromolecular drug-receptor complex concerning time, a molecular dynamics (MD) simulation for 100 ns for the macromolecular complex of ligand **MM129** with all the CDKs considered in the current research paradigm was executed. MD simulation was performed at the molecular level at a constant temperature of 300 K and constant pressure conditions. Schrödinger-Desmond (version 2019.4) module of Maestro software was used to perform dynamics simulation by using OPLS3e force field and solvating it in an explicit water box of size 10. The single point charge (SPC) model was used to infer the water molecules [100]. The usage of the OPLS3e force field and the SPC water model for macromolecular complexes with small ligands were previously described to yield the optimum reproducible results [101]. The complex was neutralized by adding the contrary-charged ions followed by their energy minimization [102]. The long-range electrostatic connections between the macromolecule and the complexed ligand were computed by applying the particle-mesh Ewald (PME) method with 0.8 grid spacing and a cutoff radius of 9.0 for Coulomb interactions after the NPT ensemble, MD simulation was run.

### 4.9. Statistical Analysis

All data are presented as the mean values with standard deviations (SD) obtained from three independent experiments. The statistical analysis was evaluated with GraphPad Prism 8.0 software system (GraphPad Prism Software Inc., San Diego, CA, USA), by using one-way ANOVA with Tukey’s test (post hoc multiple comparisons in distributions not-departing from normality). A *p*-value of <0.05 was considered significant.

## 5. Conclusions

**Figure 9:**
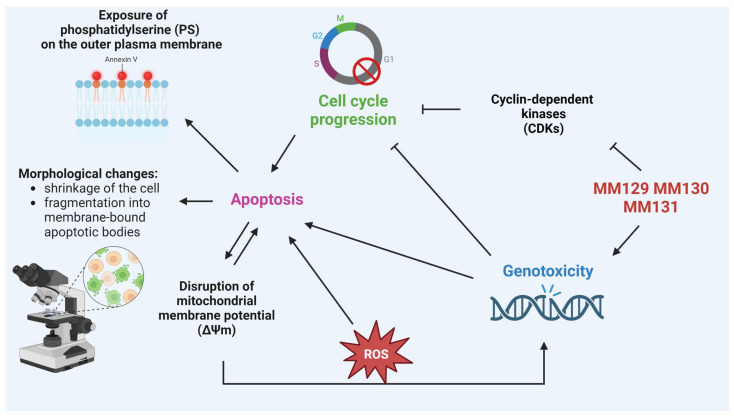
The potential mechanism of action of **MM129**, **MM130**, and **MM131**. Investigated sulfonamides cause DNA single-strand and double-strand breaks and simultaneously inhibit CDKs. High levels of DNA damage and inhibition of CDKs lead to cell cycle arrest. As the effect of high genotoxic stress and the action of the G1/S cell cycle checkpoint, the cell is directed to the apoptosis pathway. The consequences of apoptosis include cell morphological changes and the exposition of PS on the outer plasma membrane. Furthermore, apoptotic changes cause loss of Δ*Ψm* and lead to the opening of PTP, which results in ROS formation. The continuous accumulation of ROS may enhance genotoxic stress and apoptosis, leading to a self-perpetuating cycle of changes.

The presented research demonstrates the diverse, cell-line-dependent pro-oxidative and pro-apoptotic activity of novel pyrazolo[4,3-*e*]tetrazolo[1,5-*b*][1,2,4]triazine sulfonamides, such as **MM129**, **MM130**, and **MM131**, on HeLa, PC-3, HCT 116, and BxPC-3 cells. The results from the H_2_DCFDA and apoptotic assays (Annexin V binding assay, MitoTracker assay, and AO/EB) proved the high pro-oxidative and pro-apoptotic activity of **MM129**, **MM130**, and **MM131** in vitro. All investigated compounds also affected the cell cycle distribution inducing the cell-line-dependent cycle arrest in the G0/G1 or S phase. Our previous data have shown the high genotoxic potential of examined compounds toward tested cancer cells [63]. Therefore, we consider that the increase in apoptosis and the changes in the cell cycle distribution result from the strong genotoxicity of **MM129**, **MM130**, and **MM131**. Furthermore, based on computational studies, we conclude that the ability of the investigated sulfonamides to change the distribution of the cell cycle may result from their potential for CDKs inhibition. Moreover, we suggest that a high level of ROS may enhance the genotoxic and pro-apoptotic properties of the investigated triazine derivatives (Figure 9).

In conclusion, the obtained data combined with the results from our previous study [63] provide important information on the biological response of the tested cancer cell lines to **MM129**, **MM130**, and **MM131**. Moreover, our results expand the general knowledge about the antineoplastic activity of a group of MM compounds. Interestingly, among examined sulfonamides, **MM129** at the IC_50_ exhibited the highest apoptotic properties in the Annexin V binding assay against HeLa cells following the 48 h incubation period (Figure 2). Moreover, the same dose of **MM129** induced the highest accumulation of cells in the subG1 phase in the case of HeLa and PC-3 cells after the 24 h of compound exposure, compared to other triazine derivatives (Figure 7). In addition, **MM129** has presented the lowest binding energy values when docked against CDK enzymes, compared to **MM130** and **MM131** (Table 1). In contrast, **MM131** at the IC_50_ has revealed the highest pro-oxidative activity among examined sulfonamides towards all types of cancer cells following the 2 h of incubation time (Figure 6). However, our results suggest that, except for those differences, the anticancer activity of tested MM compounds seems to be relatively similar. The general comparison of the anticancer activity of the tested sulfonamides based on the IC_50_ and 24 h and 48 h incubation times is demonstrated in the Supplementary Material (Table S2). Undoubtedly, all the investigated triazine derivatives exhibit high antineoplastic properties. Nevertheless, despite the solid base, further investigations of the chemotherapeutic potential of **MM129**, **MM130**, and **MM131** towards cervical cancer, colorectal carcinoma, prostate cancer, and pancreatic adenocarcinoma, especially on in vivo material, are required.

## Figures and Tables

**Table 1:** Molecular docking results of MM compounds and CDK enzymes.

Target	PDB Code	MM129	MM130	MM131	Reference Ligand
CDK1	6gu6	−8.85	−8.06	−8.72	−8.11
CDK2	3bhu	−9.19	−8.01	−8.73	−4.63
CDK4	2w9z	−9.48	−8.03	−8.25	−7.69
CDK5	1unh	−8.78	−7.14	−7.98	−6.65
CDK6	6oqo	−8.73	−8.0	−8.91	−8.35
CDK7	1ua2	−9.84	−8.71	−9.42	−7.81
CDK8	6t41	−8.69	−7.03	−8.28	−8.01
CDK9	3blq	−8.08	−6.86	−7.0	−8.2

**Table 2:** The coordinates of the grid boxes used in the docking studies of MM compounds to currently investigated CDK enzymes.

Target	PDB Code	x-D	y-D	z-D	Spacing (Ả)	X-Center	Y-Center	Z-Center
CDK1	6gu6	60	60	60	0.503	241.389	216.129	209.535
CDK2	3bhu	40	40	40	0.375	−7.638	20.962	−21.4
CDK4	2w9z	50	50	50	0.453	20.281	25.506	8.713
CDK5	1unh	40	40	40	0.469	39.357	16.375	31.45
CDK6	6oqo	40	40	40	0.469	21.984	38.012	−9.828
CDK7	1ua2	40	40	40	0.375	41.304	−4.892	23.033
CDK8	6t41	40	40	40	0.469	−3.707	−10.927	9.279
CDK9	3blq	40	40	40	0.469	50.291	−18.937	−11.183

## Data Availability

The data presented in this study are available in the main text of this article/supplementary materials of this article or on request from the corresponding author.

## Supplementary Materials

The following supporting information can be downloaded at: https://www.mdpi.com/article/10.3390/ijms24108504/s1.

## Sample Availability

Samples of the compounds **MM129**, **MM130**, and **MM131** available from the authors.

## Abbreviations

5-FU	5-fluorouracil
AIF	apoptosis-inducing factor
AKT	Protein kinase B
AO/BE	acridine orange/ethidium bromide
ASK1	apoptosis signal-regulating kinase 1
BTK	Bruton’s tyrosine kinase
CDKs	cyclin-dependent kinases
c-FLIP	FLICE-inhibitory protein
CKIs	cyclin-dependent kinase inhibitors
CRC	colorectal cancer
DCF	fluorescent 2′,7′-dichlorofluorescein
DD	death domain
EB	ethidium bromide
ER	endoplasmic reticulum
FITC	fluorescein isothiocyanate
H_2_DCF	2′,7′-dichlorodihydrofluorescein
H_2_DCFDA	2,7-dichlorodihydrofluorescein diacetate
mTOR	mammalian target of rapamycin
MTT	3-(4,5-Dimethylthiazol-2-yl)-2,5-diphenyltetrazolium bromide
PARP	poly(ADP-ribose) polymerase
PCD	programmed cell death
PD-1	programmed cell death protein 1
PD-L1	programmed death ligand-1
PI	propidium iodide
PI3K	phosphoinositide-3-kinase
PS	phosphatidylserine
PME	particle-mesh Ewald
PTP	permeability transition pore
RMSD	root mean square deviation
RMSF	root mean square fluctuation
ROS	reactive oxygen species
SPC	single point charge
TP53	cellular tumor antigen p53
Δ*Ψm*	mitochondrial membrane potential
