# Supplementary material for: Pyrazolo[4,3-e]tetrazolo[1,5-b][1,2,4]triazine Sulfonamides as Novel Potential Anticancer Agents: Apoptosis, Oxidative Stress, and Cell Cycle Analysis
Source: Int J Mol Sci. 2023 May 9;24(10):8504. doi: 10.3390/ijms24108504 (PMC10218340; doi:10.3390/ijms24108504)
Supplement: Supplementary file 1 [file ijms-24-08504-s001.zip › ijms-2362035-supplementary.pdf]

Table S1. Cytotoxic properties of **MM129**, **MM130**, and **MM131** presented as IC_{50}. Mean values and 95% confidence intervals (parentheses) are given in μM [1].

Cell type	**MM129**	**MM130**	**MM131**
PBMCs	1.11 (0.98–1.26)	0.77 (0.71-0.84)	0.62 (0.52–0.72)
Hs27	1.15 (0.95–1.37)	0.81 (0.64–0.99)	0.62 (0.43–0.82)
HeLa	0.9 (0.73–1.06)	0.59 (0.51–0.67)	0.41 (0.28–0.54)
HCT 116	0.6 (0.58–0.62)	0.44 (0.43–0.45)	0.39 (0.37–0.41)
PC-3	0.36 (0.32–0.4)	0.22 (0.18–0.28)	0.17 (0.16–0.17)
BxPC-3	0.26 (0.2–0.32)	0.17 (0.13–0.21)	0.13 (0.11–0.15)

1. Flow Cytometry Assessment of Annexin V Binding

Figures S1-S12 show the exemplary flow cytometry (Annexin V-FITC/PI assay) dot-plots for depicting apoptotic and necrotic changes in examined cancer cell lines (Hela, HCT 116, PC-3, and BxPC-3) exposed to **MM129**, **MM130,** and **MM131** in concentrations of ½ IC_{50}, IC_{50}, and 2×IC_{50} for 24-h, 48-h, and 72-h. Q1 (upper left quadrant) – necrotic cells (Annexin V-, PI+); Q2 (upper right quadrant) – late apoptotic cells (Annexin V+, PI+); Q3 (lower right quadrant) – early apoptotic cells (Annexin V+, PI-); Q4 (lower left quadrant) – live cells (Annexin V-, PI-).

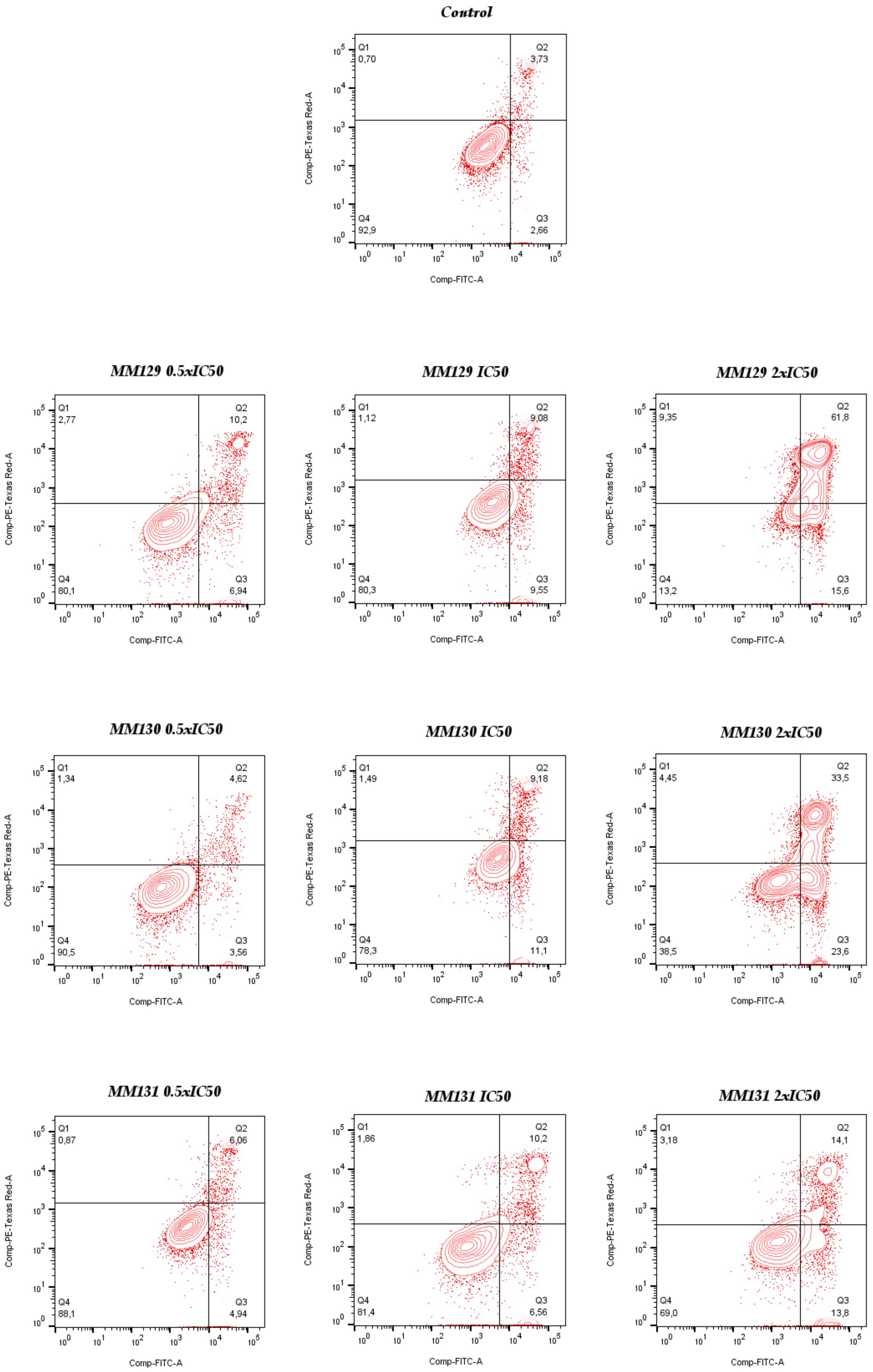

Figure S1. Hela cells after 24-h of exposure to MM-compounds.

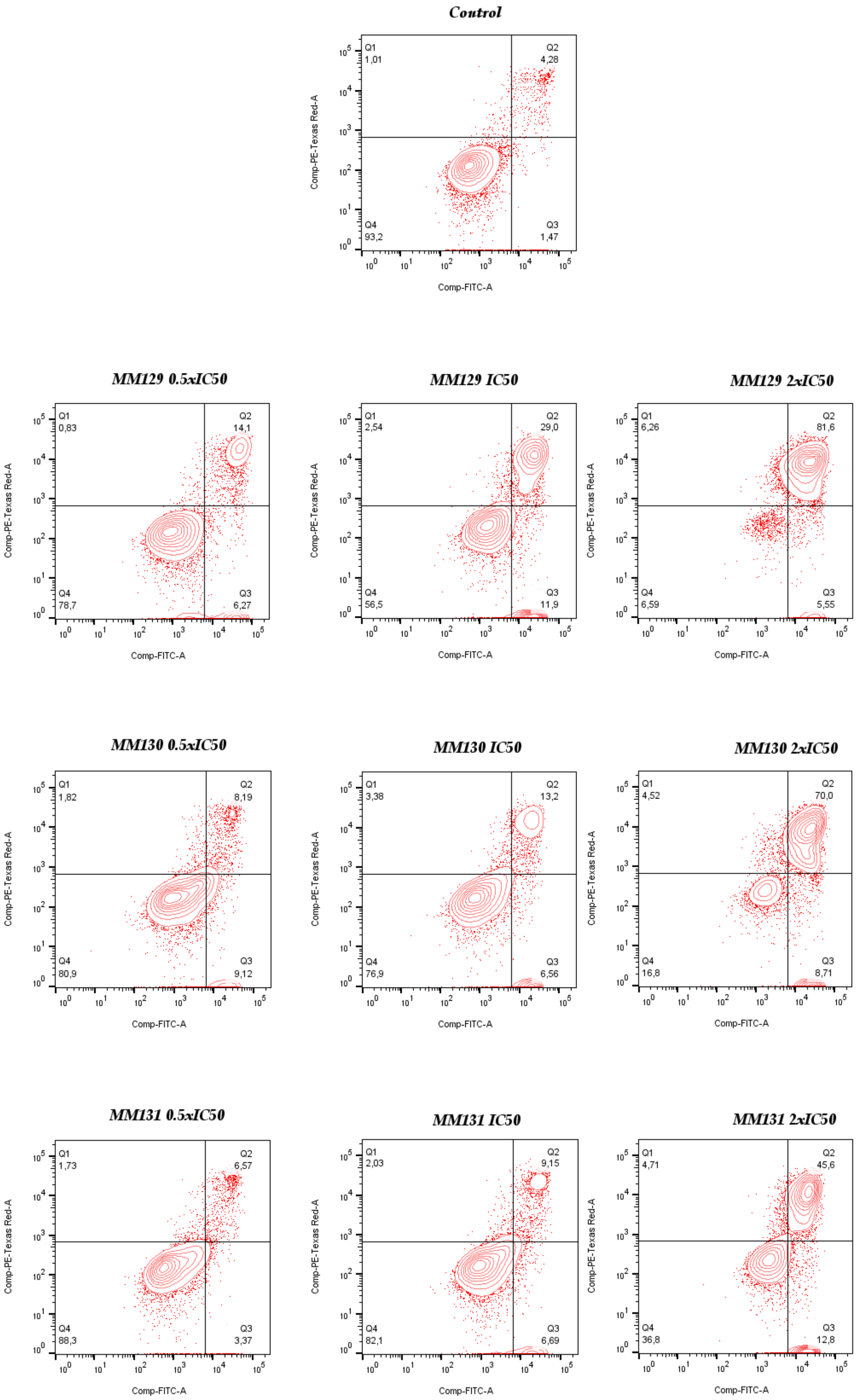

Figure S2. Hela cells after 48-h of exposure to MM-compounds.

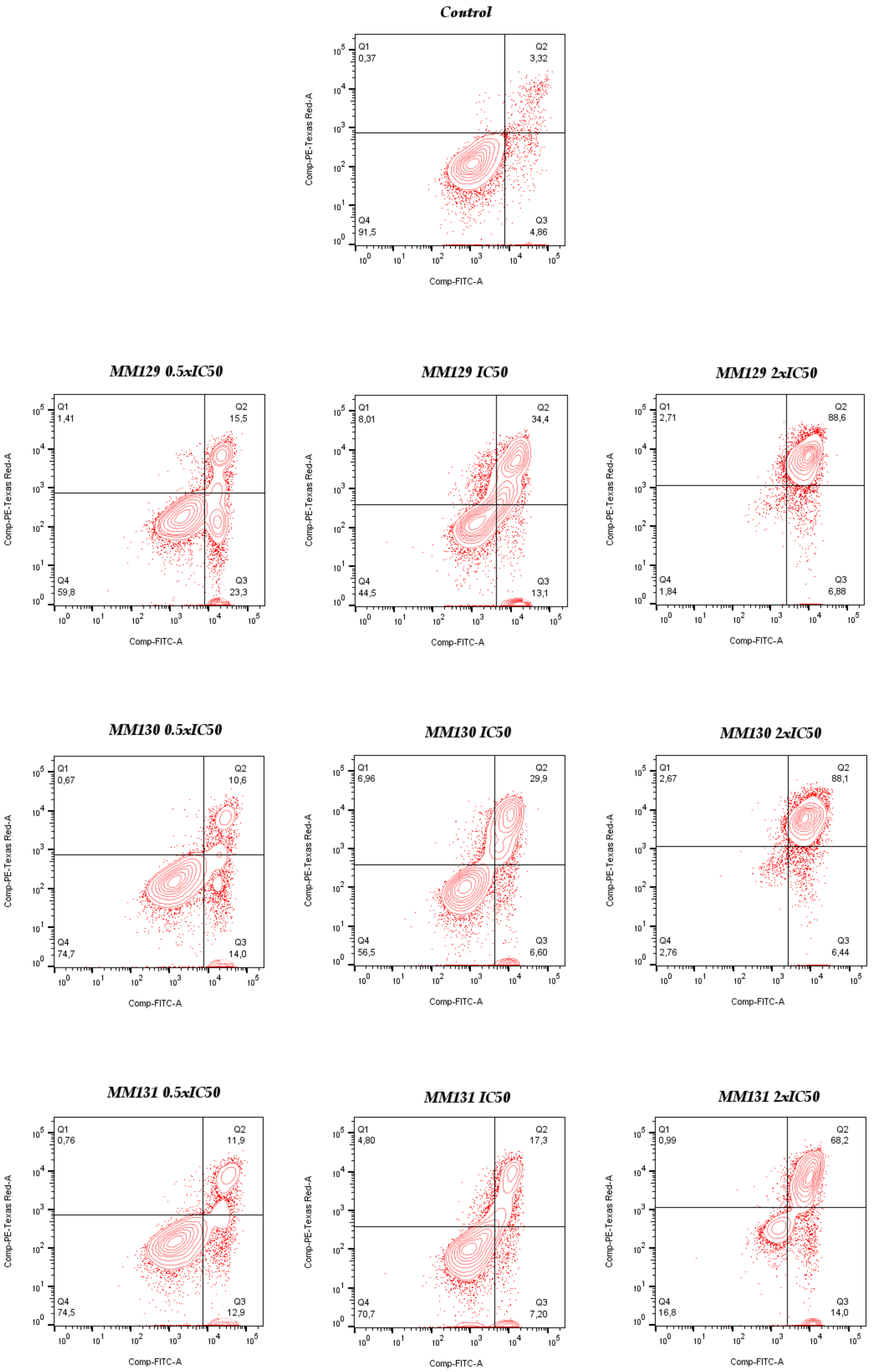

Figure S3. Hela cells after 72-h of exposure to MM-compounds.

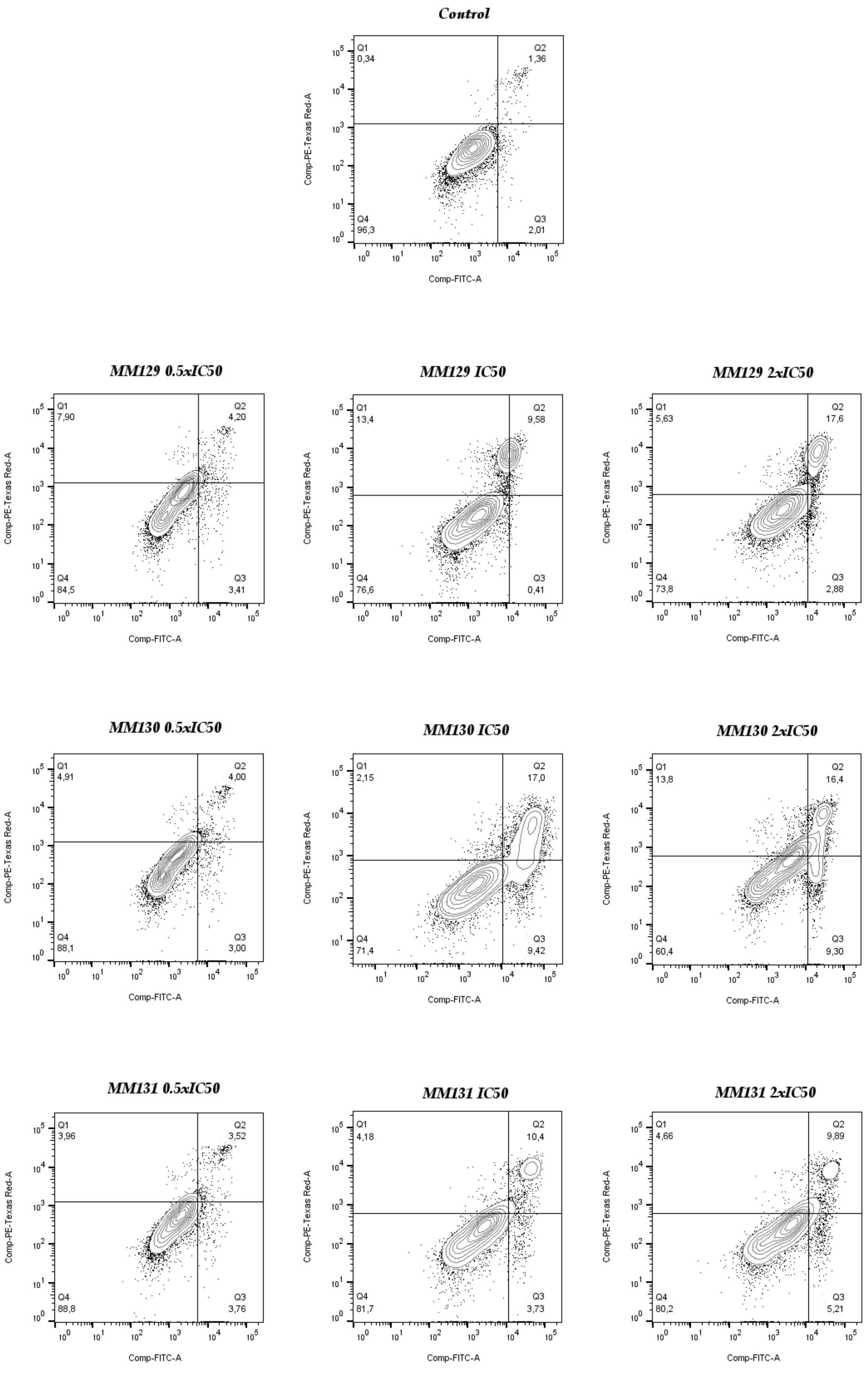

Figure S4. HCT 116 cells after 24-h of exposure to MM-compounds.

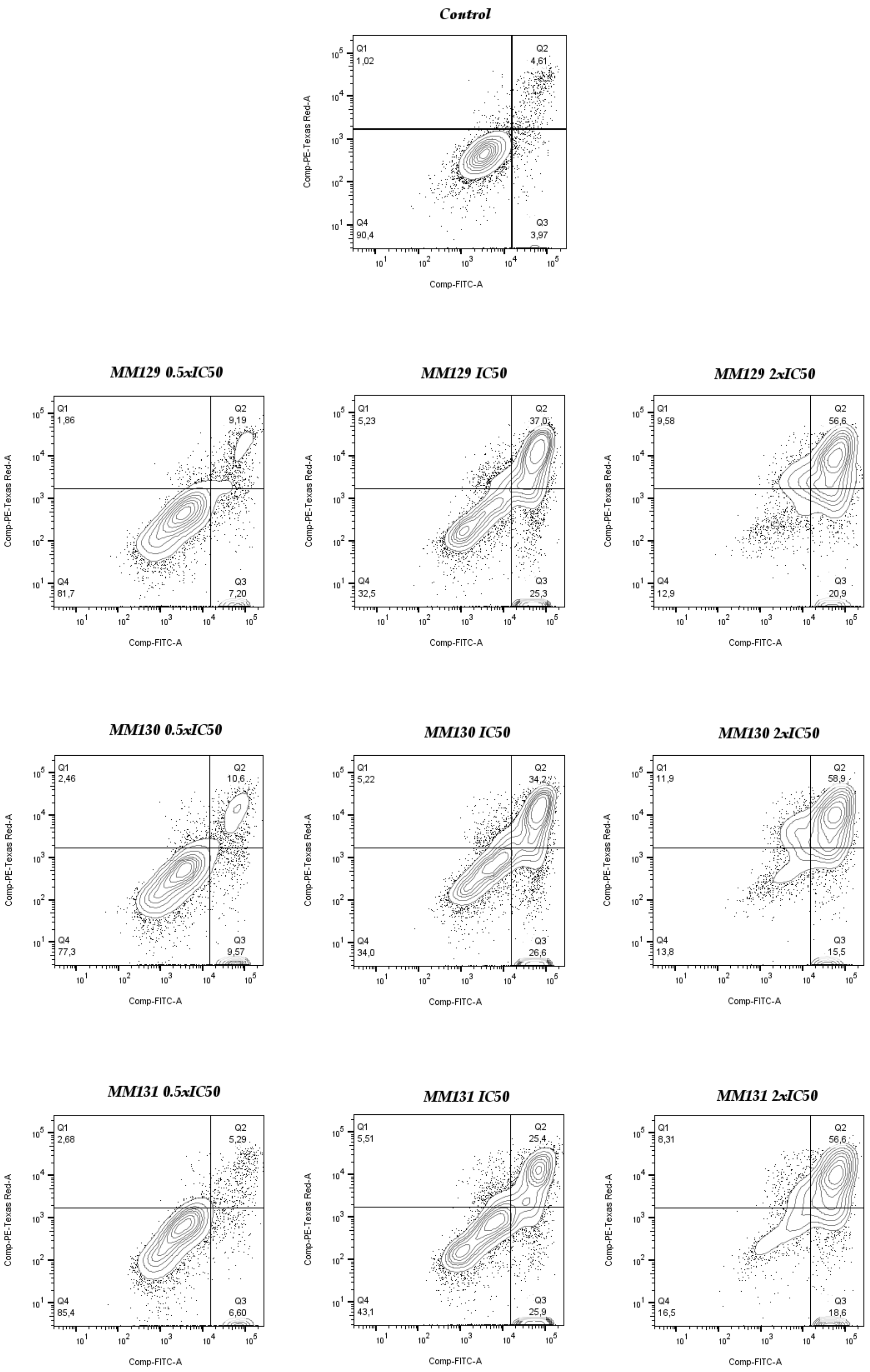

Figure S5. HCT 116 cells after 48-h of exposure to MM-compounds.

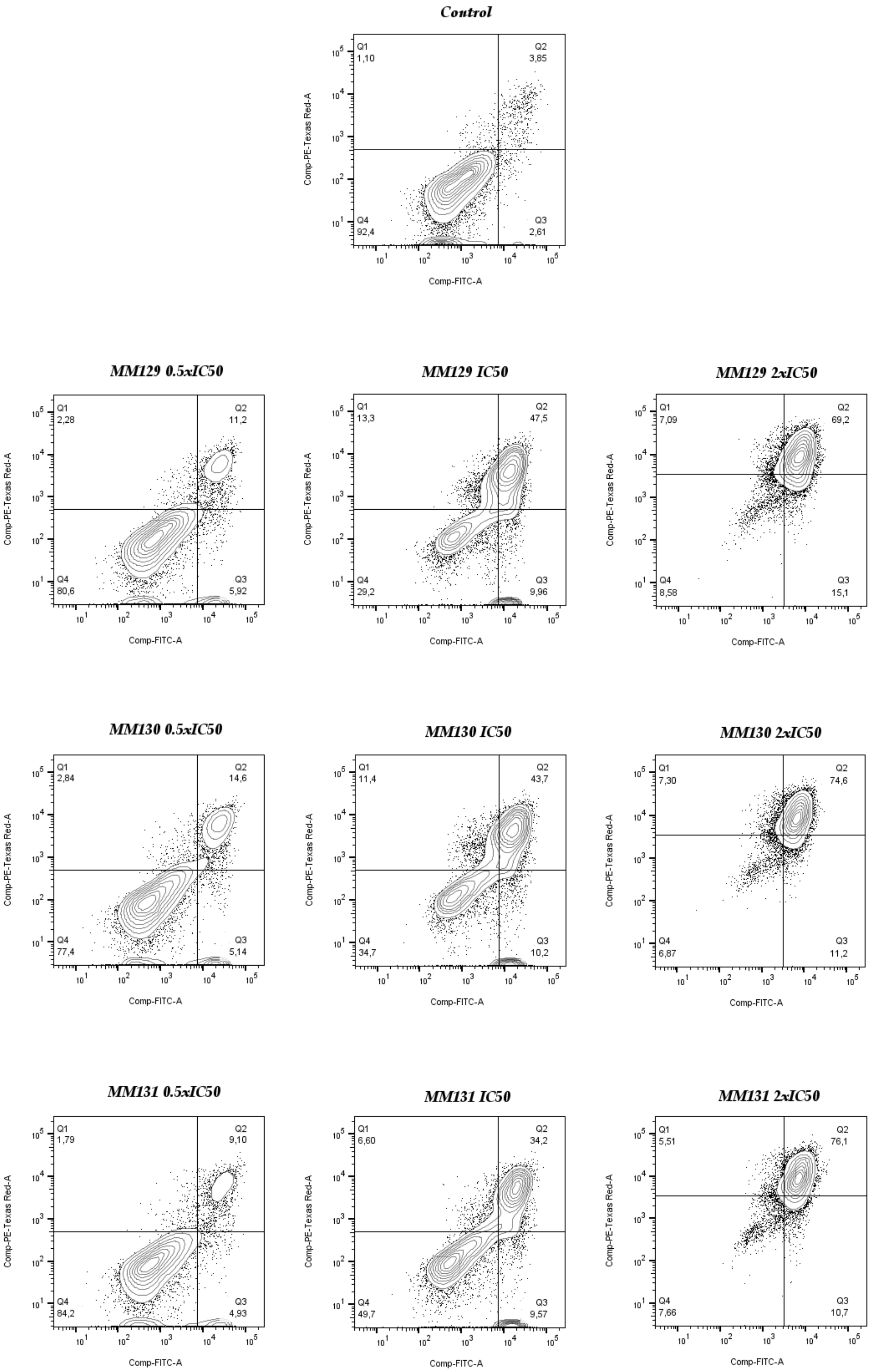

Figure S6. HCT 116 cells after 72-h of exposure to MM-compounds.

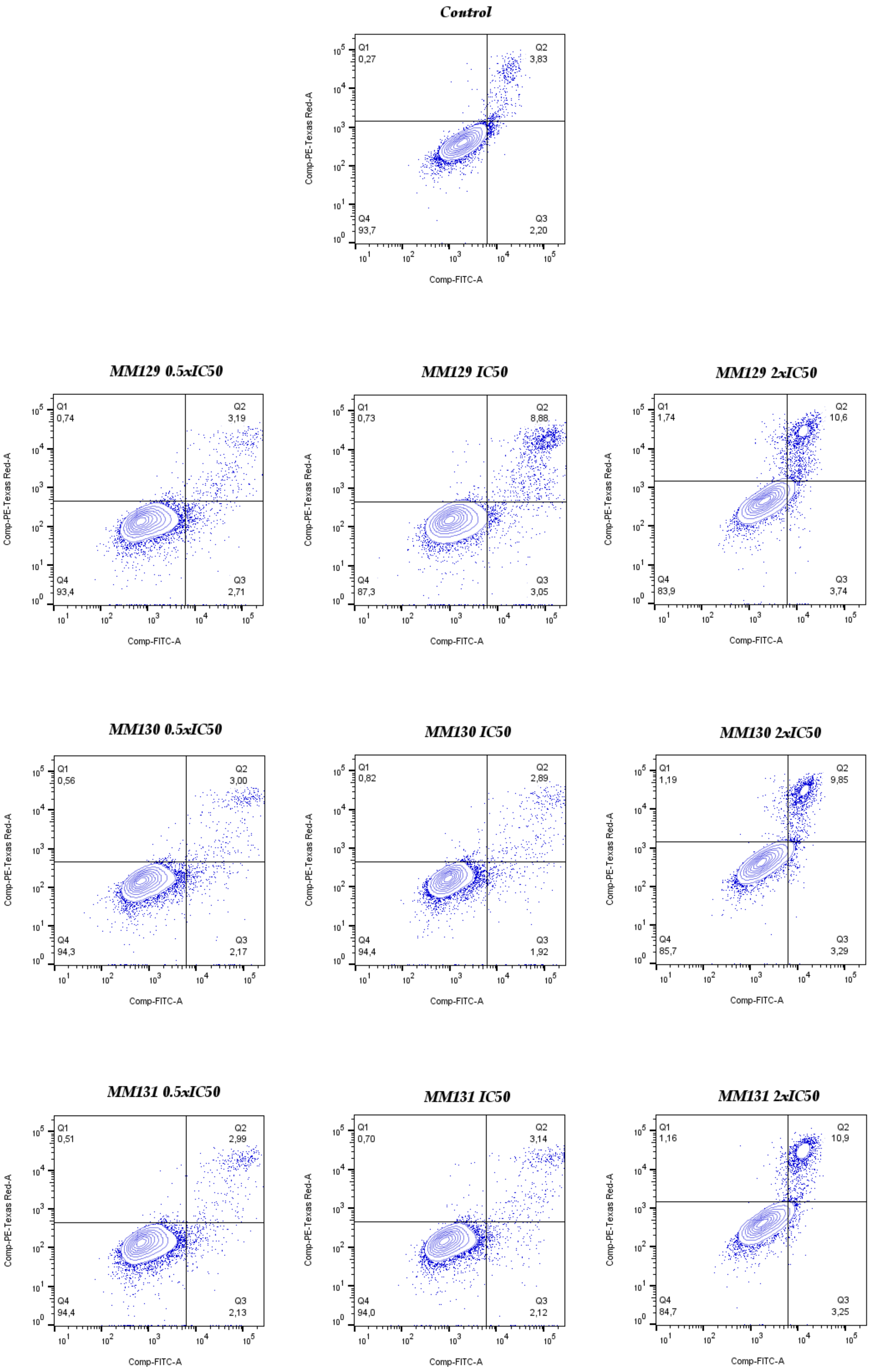

Figure S7. PC-3 cells after 24-h of exposure to MM-compounds.

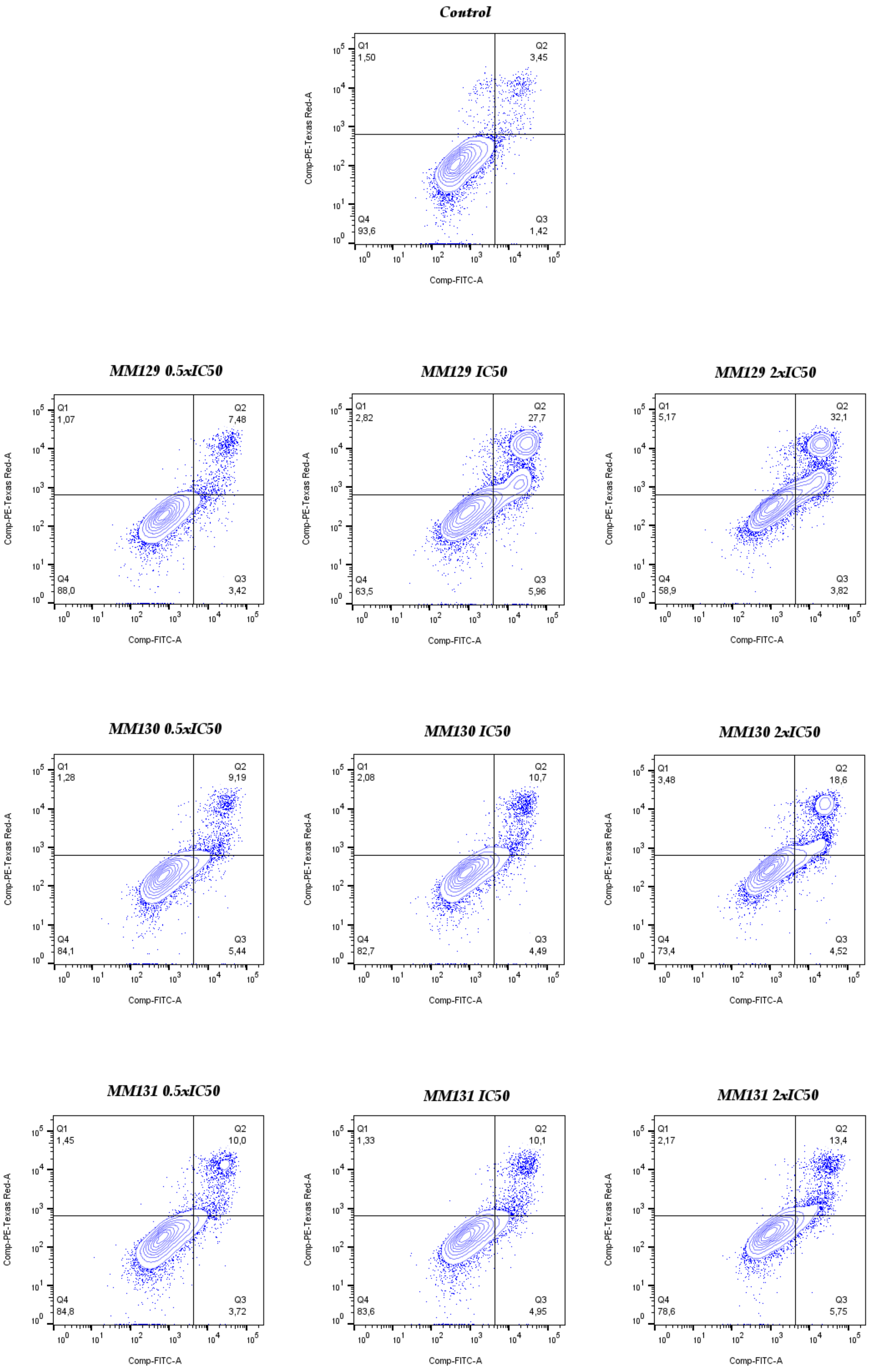

Figure S8. PC-3 cells after 48-h of exposure to MM-compounds.

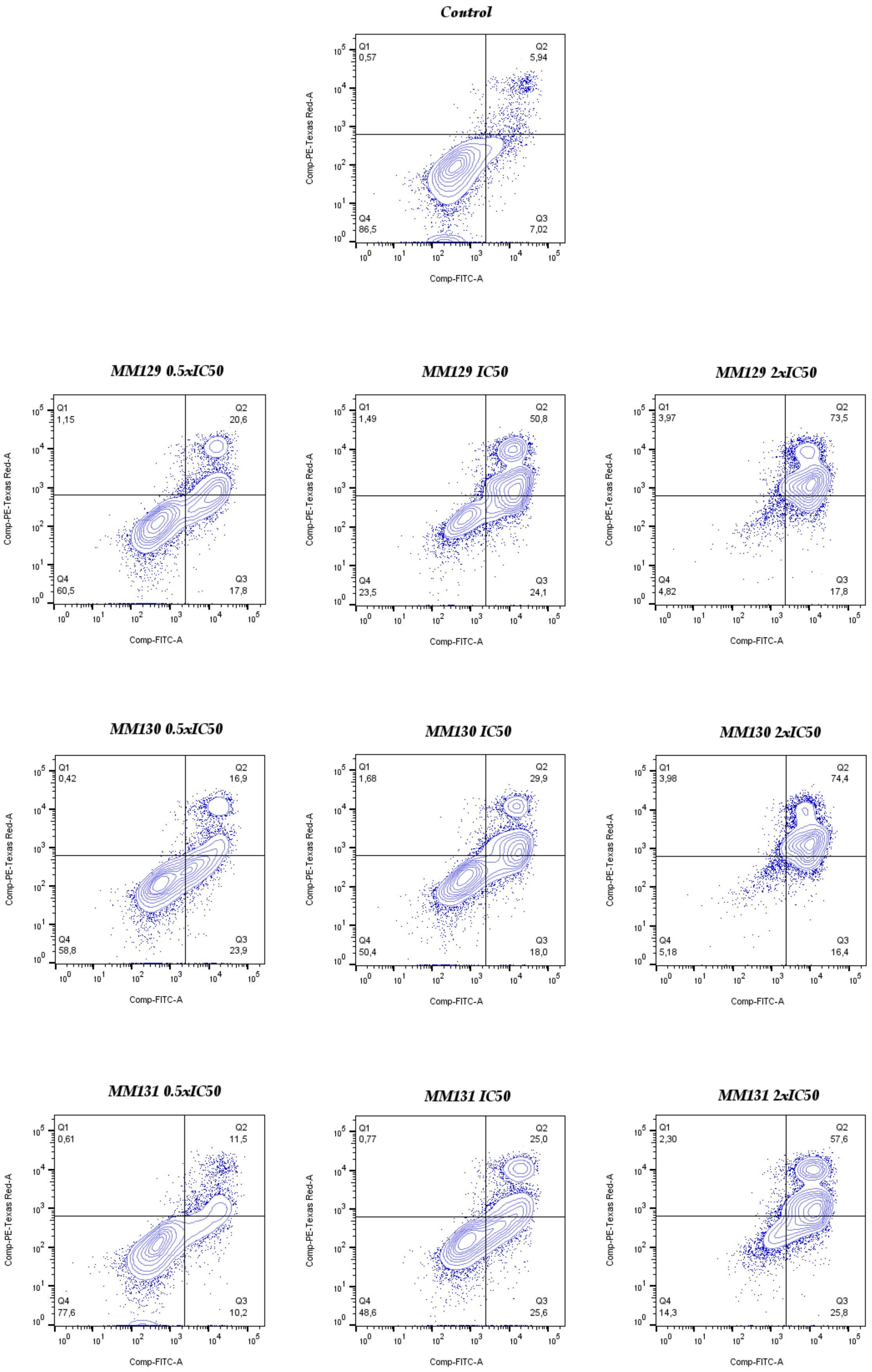

Figure S9. PC-3 cells after 72-h of exposure to MM-compounds.

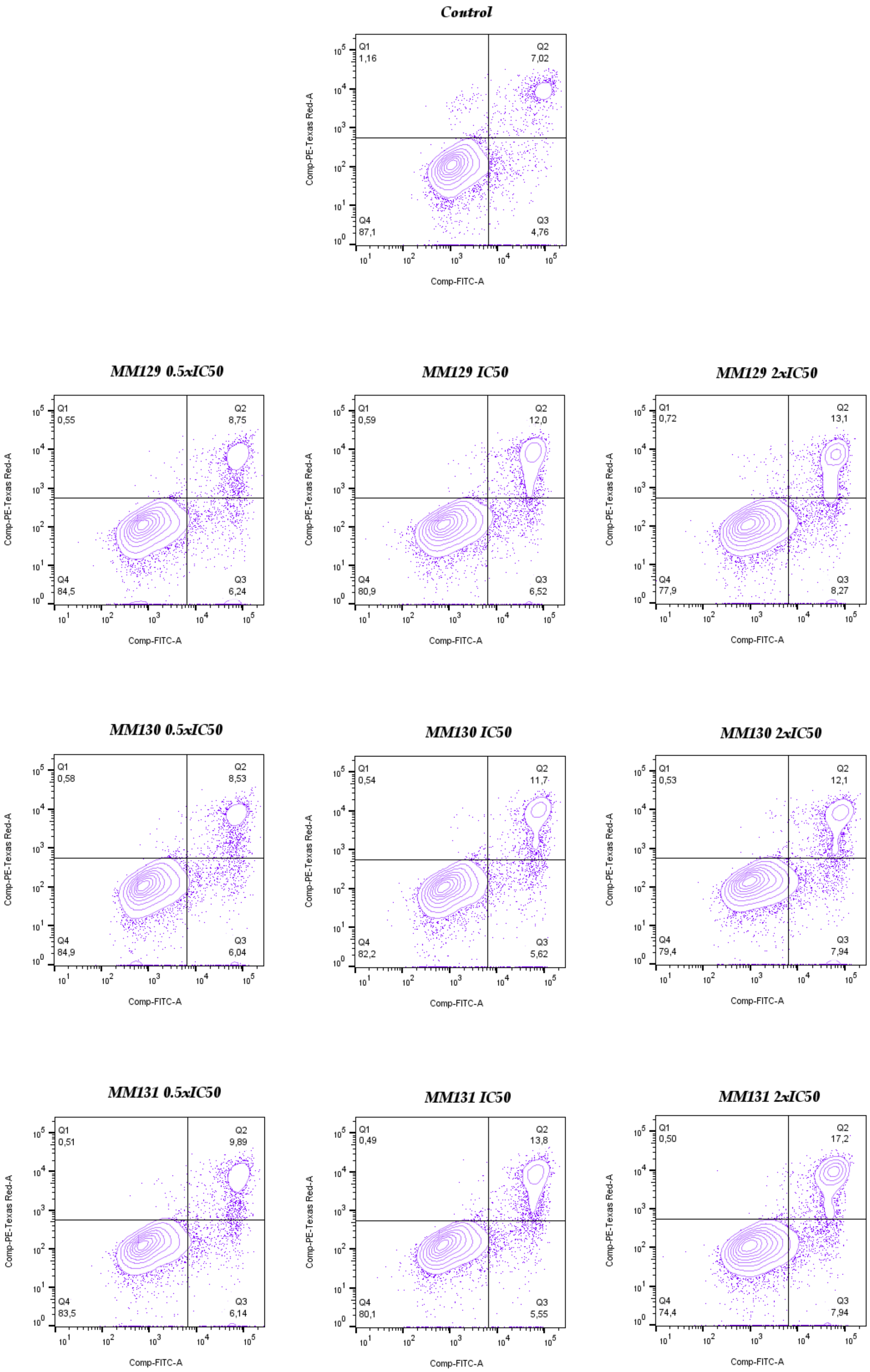

Figure S10. BxPC-3 cells after 24-h of exposure to MM-compounds.

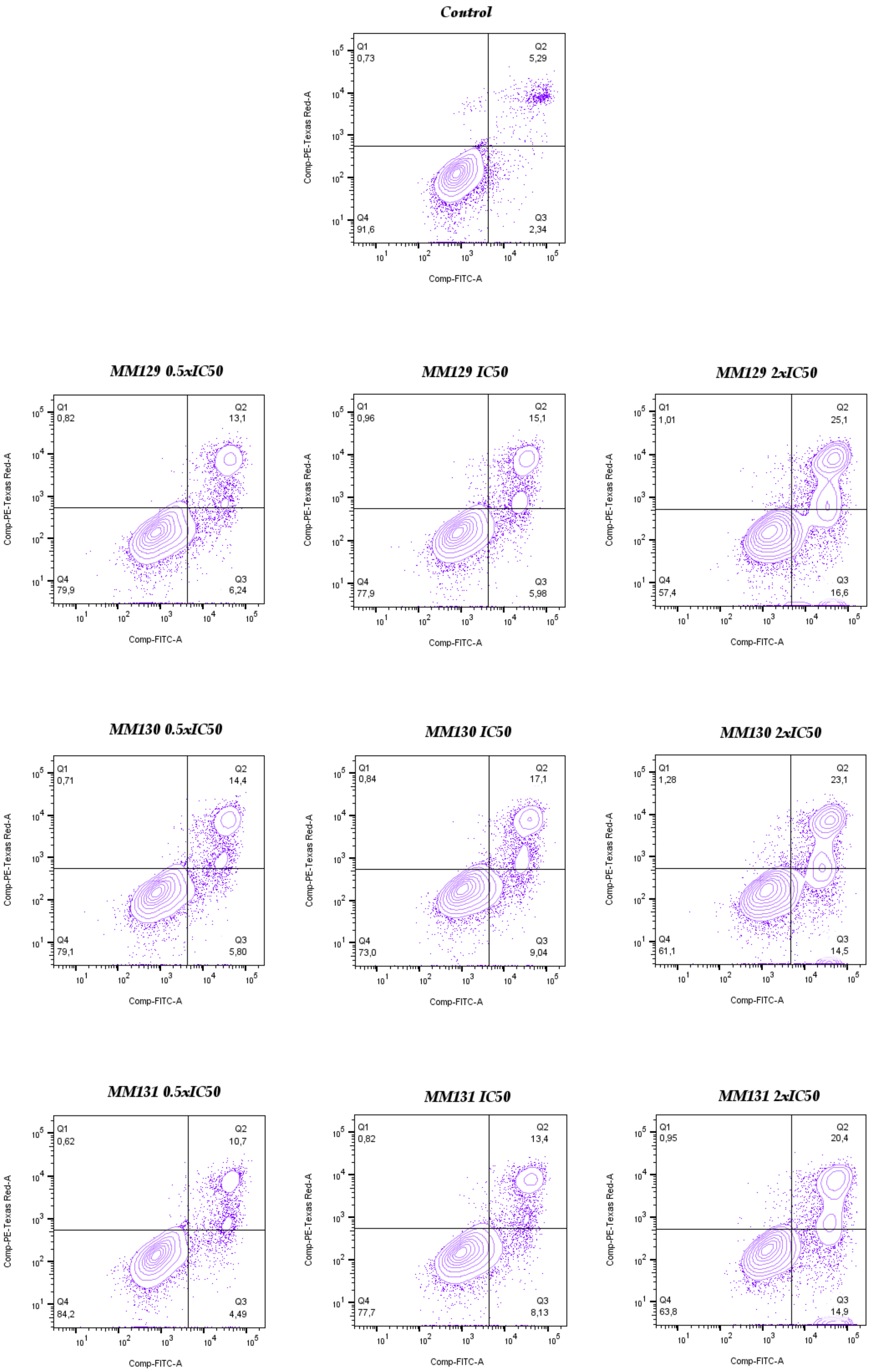

Figure S11. BxPC-3 cells after 48-h of exposure to MM-compounds.

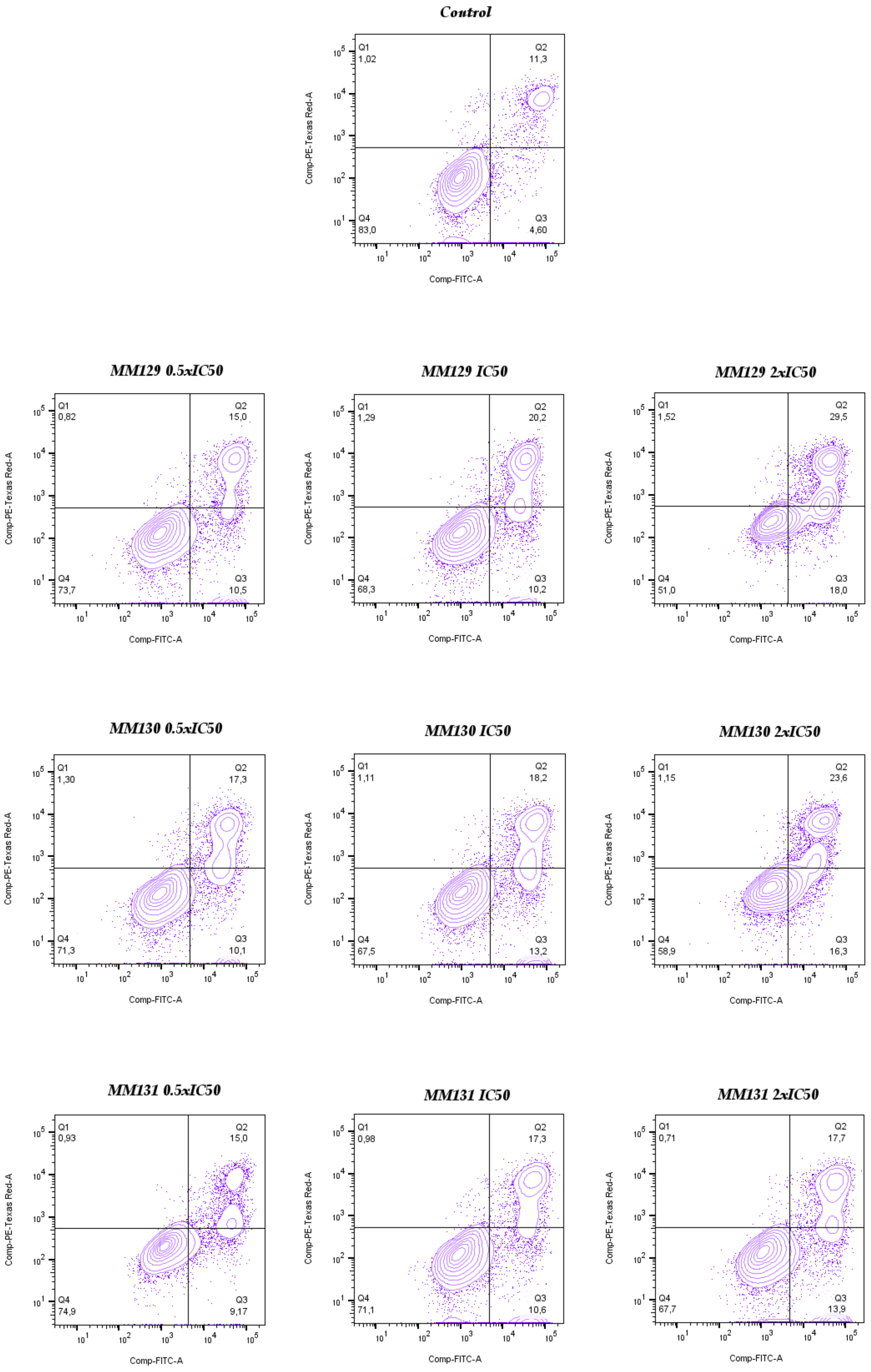

Figure S12. BxPC-3 cells after 72-h of exposure to MM-compounds.

2. Cell cycle analysis

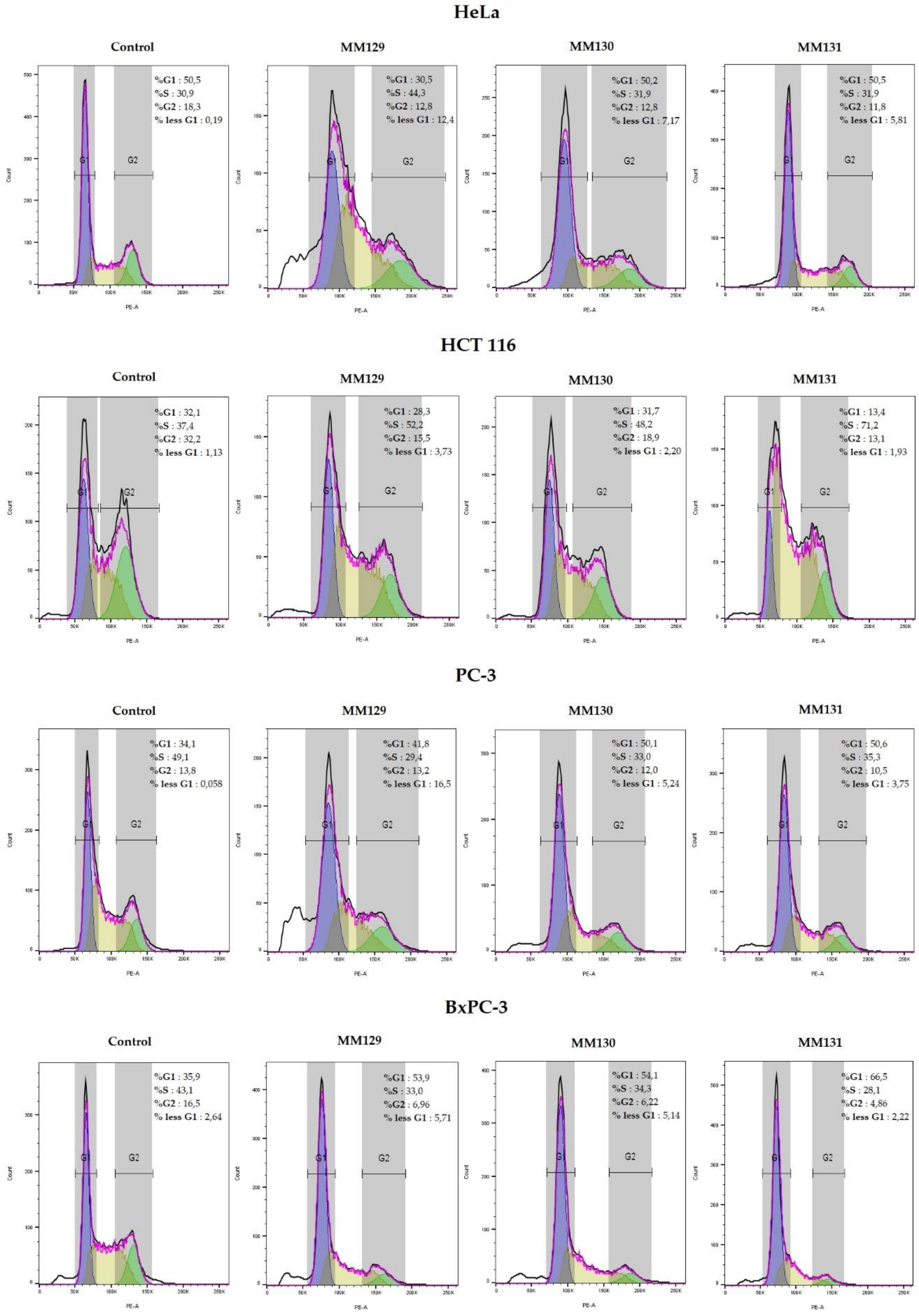

Figure S13. Representative dot-plots presenting the cell cycle distribution of HeLa, HCT 116, PC-3 and BxPC-3 cell lines after their 24-h incubation with **MM129**, **MM130**, and **MM131** in the concentration of IC_{50}.

Table S2. Comparison of anticancer activity of **MM129**, **MM130**, and **MM131** in the concentration of IC_{50} toward HeLa, HCT 116, PC-3, and BxPC-3 cancer cells following 24-h and/or 48-h treatment. Significant differences (p < 0.05) compared with control (bold and *). MM compound that exhibited the highest activity (significant differences p < 0.05) among investigated sulfonamides in the given assay is blue color-coded.

Method	Exposure time	Cell line	Control	MM129	MM130	MM131
Annexin V binding [1]	24-h	HeLa	6.7 ± 0.7	**17.4 ± 1.7 ***	**17.3 ± 2.6 ***	**15.5 ± 1.4 ***
		HCT 116	6.9 ± 2.6	**14.4 ± 2.7 ***	**16.8 ± 6.3 ***	**14.3 ± 3.3 ***
		PC-3	9 ± 2.8	9 ± 3.4	5.9 ± 2.2	6.2 ± 2.7
		BxPC-3	13.8 ± 1.8	17.1 ± 1.2	18.1 ± 3.2	18.5 ± 1.3
	48-h	HeLa	7.1 ± 1.8	**40 ± 0.82 ***	**20.6 ± 1.7 ***	**15.9 ± 0.66 ***
		HCT 116	10.4 ± 2.3	**61 ± 1.1 ***	**57.3 ± 3 ***	**50.2 ± 1 ***
		PC-3	6.7 ± 1.6	**23.3 ± 9 ***	14.3 ± 0.8	14.4 ± 1
		BxPC-3	12 ± 3.4	**19.4 ± 3.3 ***	**24.8 ± 4.4 ***	**21.2 ± 1.9 ***
MitoTracker Red CMXRos [2]	24-h	HeLa		85.5 ± 7.6	87.6 ± 2.2	87.5 ± 0.9
		HCT 116		**83.6 ± 2.8 ***	88.6 ± 3.5	85.4 ± 1.8
		PC-3		91.7 ± 8.5	91.2 ± 6.7	92.1 ± 1.4
		BxPC-3		90.5 ± 3.1	87.3 ± 3.7	89.8 ± 7
	48-h	HeLa		86.5 ± 5.3	88 ± 1.3	86.9 ± 6.9
		HCT 116		**80.2 ± 7.9 ***	**82.8 ± 5.2 ***	85.3 ± 2.3
		PC-3		80.2 ± 10.8	86.6 ± 2.1	83.2 ± 3.5
		BxPC-3		84.5 ± 8.1	87.4 ± 1.6	85.4 ± 8.7
AO/EB dual staining [1]	48-h	HeLa	1.3 ± 0.6	**32 ± 6.1 ***	**23.3 ± 2.5 ***	**13.3 ± 1.5 ***
		HCT 116	2.3 ± 2.1	**23.3 ± 5.5 ***	**14.3 ± 4.5 ***	**21 ± 7.2 ***
		PC-3	6 ± 2	8.7 ± 1.5	11.7 ± 1.5	7 ± 3
		BxPC-3	3 ± 2	**16.3 ± 3.5 ***	**13 ± 2.6 ***	**14.7 ± 2.3 ***
Cell cycle [3]	24-h	HeLa		↑ subG1; ↓ G0/G1	—	—
		HCT 116		↑ S; ↓ G2/M	↑ S; ↓ G2/M	↑ S; ↓ G2/M; ↓ G0/G1
		PC-3		↑ subG1; ↓ S;	↑ subG1; ↓ S; ↑ G0/G1	↑ subG1; ↓ S; ↑ G0/G1
		BxPC-3		↑ G0/G1; ↓ G2/M	↑ G0/G1; ↓ G2/M	↑ G0/G1; ↓ G2/M

1 Percentage of apoptotic cells shown as the mean ± SD.
[2] Changes in $\Delta\Psi m$ presented as a mean ± SD relative to the control group normalized to 100%.
[3] A significant (p<0.05) increase (↑) or decrease (↓) in the percentage of cells in a particular cell cycle phase.

References:

1. Bukowski, K.; Marciniak, B.; Kciuk, M.; Mojzych, M.; Kontek, R. Pyrazolo[4,3-e]Tetrazolo[1,5-b][1,2,4]Triazine Sulfonamides as Novel Potential Anticancer Agents: Cytotoxic and Genotoxic Activities In Vitro. *Molecules* **2022**, *27*, 3761, doi:10.3390/molecules27123761.
